# Affinity on Demand: A One-Pot Method for Synthesis and Sample Enrichment Using TentaGel-Functionalized Resins

**DOI:** 10.1021/acsomega.5c02738

**Published:** 2025-04-22

**Authors:** Michalina Zawadzka, Wojciech Gil, Andrzej Konieczny, Kornelia Krakowska-Jura, Monika Kijewska, Piotr Stefanowicz

**Affiliations:** † Faculty of Chemistry, University of Wrocław, Joliot-Curie 14, Wrocław 50-383, Poland; ‡ Department of Nephrology and Transplantation Medicine, Wrocław Medical University, Borowska 213, Wrocław 50-556, Poland

## Abstract

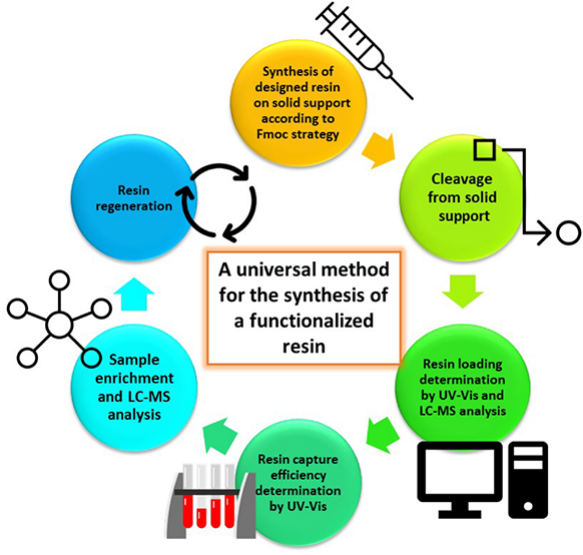

Protein glycation is a nonenzymatic reaction that results in the formation of early glycation products, commonly referred to as Amadori products, which play an important role in diabetes complications. In proteomic research, the analysis of glycated peptides is very challenging due to the low amount of analyte in a biological sample. One of the methods to overcome this is selective enrichment of the sample in the desired analyte. A method for synthesizing functionalized resins with phenylboronic acids has been developed, which allows for the incorporation of different linkers and a variable number of phenylboronic acid moieties, as well as the use of any solid support. Furthermore, the resins are prepared for use in sample enrichment following the completion of the synthesis process and demonstrate a high affinity for glycated peptides. The highest-affinity resin (4PhB-3Lys-TGR) was applied to artificially glycated albumin hydrolyzate and patient serum, and, in addition, it was used in conjunction with a biological sample (i.e., milk) for the selective enrichment of glycated peptides. The bioinformatics analysis provided results that confirmed the high coverage of protein sequences identified in the complex samples based on glycated peptides. This paper presents a novel, fast, simple, and cost-effective one-pot method for the synthesis of functionalized resins, along with a selective method for the enrichment of samples with glycated peptides. We believe that the presented approach is general and, with necessary modifications, could be applied for affinity-based isolation not only of Amadori products but also of carbonyl compounds, thiols, compounds with chelating properties, and others.

## 1 Introduction

Proteomics is the qualitative and quantitative analysis of all proteins involved in specific biochemical pathways in cells, tissues, or organs.^1,2^ The study of differences in protein profiles, including changes in their concentration between physiological and pathological states of tissues or cells, can lead to the identification of proteins associated with the development of a given disease, including potential biomarkers and metabolic pathways with a hitherto unknown role in pathophysiology.^3−5^ In recent years, proteomics has been a rapidly developing scientific field^6^ made possible by technological advances, including access to modern, fully automated mass spectrometers coupled with ultrahigh-performance liquid chromatography (UHPLC-MS)^7^ and bioinformatics databases.^8^ The main problem associated with the study of complex biological systems is often the lack of sensitivity of the analysis, which prevents the identification of some proteins, especially post-translationally modified proteins, present in too small amounts of the sample taken for analysis.^9^ Proper preparation of biological material for testing becomes a real analytical challenge.^10^ Improving analytical sensitivity can be done in two ways: (i) improving the ionization of the analyte by introducing a stable charge^11−13^ or (ii) using techniques to enrich the sample only for specific analytes by removing the matrix.^14,15^ The implementation of the second approach requires the design and synthesis of a functionalized resin that selectively interacts with the selected compounds and the optimization of the detection procedure to eliminate the matrix effect (interference effect), making it impossible to analyze the analyte in the presence of other compounds in the matrix.^16^ The use of sample concentration methods increases the chance of identifying new compounds present in trace amounts, which can enable early detection of lesions by detecting molecular biomarkers of specific pathological conditions in the body.^17^

In recent years, there has been a notable increase in interest in boronate affinity materials (BAM), which have been the subject of intense development and are being employed in a growing number of applications.^18^ BAM is a widely utilized adsorbent for *cis*-diol-containing molecules, with the properties of covalent reversible binding between boronic acid and cis-diols. Analysis has been taken to a new level through the use of different types of boronate ligands and functional materials combined with advanced technologies.^19^ The most common approach to enhancing the capabilities of boronic acids for glycopeptide and glycoprotein enrichment is to immobilize them onto a range of solid supports, including MOFs, magnetic nanoparticles, and graphene. Similarly, lectins are typically immobilized onto solid supports, such as agarose or magnetic beads.^19^ Research and development have been conducted into the use of BAM sensors for specifically recognizing and detecting glycoproteins, which play a pivotal role in biological functions.^20,21^ Additionally, TentaGel resin (TGR) functionalized by lectins^22,23^ or boronic acids^24^ has been employed for the detection and enrichment of glycoproteins or glycopeptides. The detection of peptide Amadori products is of particular interest since these are early glycation products with diagnostic potential. Consequently, studies are being conducted that focus on sugar-phenylboronic acid and Amadori product-phenylboronic acid interactions, as well as their ester formation and stability.^25^ The use of phenylboronic acids has also been employed in the selective detection of *cis*-diol compounds by ESI-MS. The ammonium salts of phenylboronic acids have been shown to form a conjugate that increases the ionization efficiency of both sugars and Amadori products in mass spectrometry.^26^ The enrichment of samples in Amadori products is commonly achieved through the utilization of functionalized agarose with *m*-aminophenylboronic acid.^27,28^ However, to the best of our knowledge, linker-modified TentaGel resin in conjunction with a phenylboronic acid moiety has not previously been employed for the selective capture of glycated peptides for subsequent LC–MS analysis.

A modern lifestyle disease is diabetes,^29^ in which Schiff bases are formed between glucose and the side groups of lysine residues in the early stages, which further regroup to form the Amadori product.^30^ Uncontrolled hyperglycemia leads to the deposition of early glycation products as well as the formation of advanced glycation products as a result of further reactions (oxidation, cross-linking), which severely impair organs such as the kidneys, eyes, and blood vessels.^31^ Over the past few years, glycated albumin (GA) has gained increasing interest as a new biomarker for diabetes mellitus (DM) in clinical settings.^32−34^

GA reflects changes in blood glucose concentration over a period of 2–3 weeks due to its lifespan of 21 days and is not affected by hematologic disorders.^35^ In addition to GA, other glycated proteins have potential as biomarkers for diabetes. Proteins such as alpha-2-macroglobulin, beta-2-glycoprotein 1, apolipoprotein A1, coagulation factor XIII A chain, and complement C4-A undergo a glycation reaction and have been found in the blood plasma of DM patients.^36,37^

Amadori products are also observed during food processing. The heat treatment, processing, or storage of low-lactose milk has been demonstrated to promote the formation of Maillard products, a consequence of the high content of reducing sugars and proteins in the milk. In low-lactose milk, the presence of glucose and galactose has been shown to promote heightened reactivity toward the amino groups of proteins and free amino acids, resulting in the formation of the Amadori product. Such modification has the potential to alter the physicochemical properties of milk proteins and reduce the nutritional value of the milk.^38,39^ The most abundant modified milk proteins are β-lactoglobulin, α-lactoglobulin, and κ-casein, with over 60% of their free lysine residues being promoted for glycation, as identified by mass spectrometry techniques.^38,40,41^ Scientific studies correlating the degree of glycation in plasma-abundant proteins with the stage of disease, as well as searching for Amadori products in milk products, have led to the development of a substrate for the selective concentration of glycated peptides, removing the complex matrix.^42,43^

**Figure 1:**
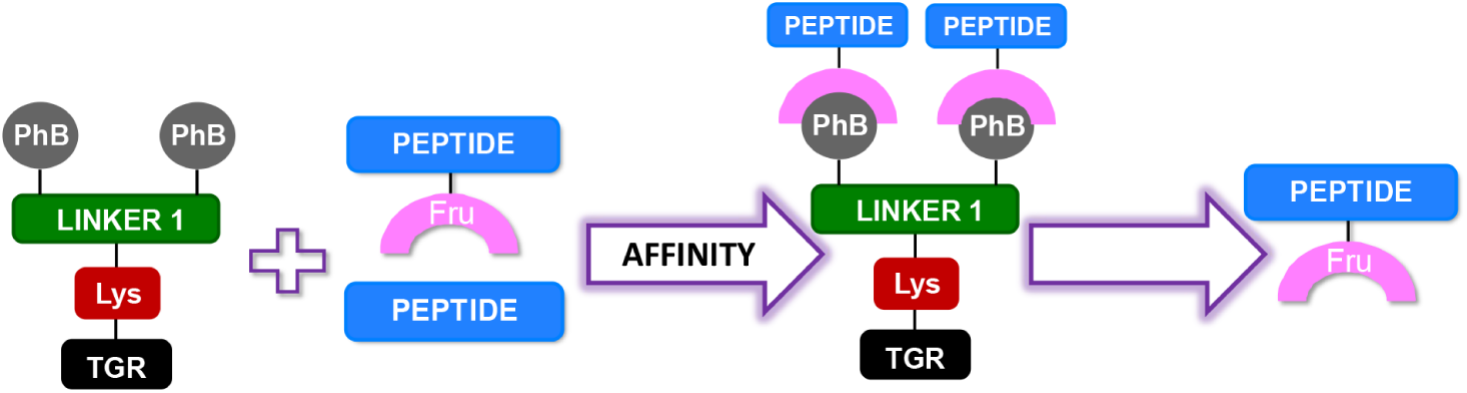
General idea of selectivity of functionalized resin toward glycated peptides.

Commercially available agarose resin functionalized with *m*-aminophenylboronic acid residues is used to concentrate the sample into glycated peptides.^44,45^ The disadvantage of this resin is that it only works in aqueous systems, has low chemical and mechanical stability, and chemical modification of this material is difficult. In contrast to the typical resin used for solid-phase synthesis, agarose presents certain difficulties with regard to modification, incorporation of new linkers, and monitoring of reaction progress. The ChemMatrix resin, cross-linked with ethylene glycol units, can be used in a similar manner. This resin was originally designed for synthesis on a solid support. This resin, functionalized with two units of phenylboronic acid derivatives, works in aqueous–organic systems, extending its application to more hydrophobic systems.^16^ Inspired by the high affinity of the functionalized ChemMatrix resin and knowing that it has recently been withdrawn from sale, in this work, we aim to present a versatile one-pot method for the synthesis and selective sample concentration of Amadori peptide products on functionalized TentaGel-type substrates without any additional purification step (Figure 1).

In this work, we offer a ready-made procedure for the synthesis of functionalized resin, showing the possibility of using any linkers to which phenylboronic acid derivatives are attached. As a solid support, we selected TentaGel resin, which is also a poly(ethylene glycol)-based resin. The TentaGel resin swells well in both polar and nonpolar solvents.^46^ Furthermore, it exhibits high chemical and mechanical resistance with low resin loading. These properties are ideal for organic synthesis on solid support followed by bioassay in aqueous media, making TGR the optimal matrix for a one-pot method for functionalized resins. Therefore, we (i) synthesized the functionalized resins using different linkers, (ii) determined the loading of the resins by UV–vis, and (iii) investigated the efficiency of the capture process with a model peptide by UV–vis and LC-MS analysis.

## 2 Experimental Section

### 2.1 Reagents

The derivatives of amino acids for peptide synthesis and the coupling reagent benzotriazole-1-yl-oxy-tris-pyrrolidino-phosphonium hexafluorophosphate (PyBOP) were purchased from Novabiochem (Darmstadt, Germany). The derivatives for functionalized resin synthesis and trifluoroacetic acid (TFA) were purchased from Iris Biotech GmbH (Marktredwitz, Germany). The TentaGel R RAM resin (0.18 mmol/g) was purchased from Rapp Polymere (Tuebingen, Germany). The solvents for peptide synthesis (analytical grade) were obtained from VWR Chemicals (Radnor, PA, USA) (*N,N*-dimethylformamide, DMF; dichloromethane, DCM) and J.T. Baker (Radnor, PA, USA) (methanol, acetonitrile). LC–MS solvents (water, acetonitrile, and methanol) were purchased from ChemSolve (Łódź, Poland) and J.T. Baker (Radnor, PA, USA). The other reagents were purchased from Sigma-Aldrich (Darmstadt, Germany): *N,N*-diisopropylethylamine (DIEA), *N,N*′-diisopropylcarbodiimide (DIC), triisopropylsilane (TIS), sodium 2-mercaptoethanesulfonate (MESNa), Fmoc-β-Ala-OH, 4-carboxyphenylboronic acid, and bromoacetic acid. Albumin from human (lyophilized powder, essentially globulin-free, ≥ 99% (agarose gel electrophoresis)) and trypsin (from bovine pancreas, lyophilized powder) were purchased from Aldrich (Darmstadt, Germany).

### 2.2 LC–UV–MS Analysis

All LC–MS experiments were conducted on LCMS–IT–TOF Shimadzu, SHIM-POL A.M., Warsaw, Poland (interface voltage: 1.70 kV, block heater temperature: 200 °C, drying gas: nitrogen) or LCMS-9030 Shimadzu, SHIM-POL A.M., Warsaw, Poland (interface voltage: 4.00 kV, block heater temperature: 350 °C, drying gas: nitrogen), equipped with an electrospray ion source, operating in either positive or negative ion mode. For LC–UV analysis (Nexera XR LC-20AD Shimadzu, SHIM-POL A.M., Warsaw; flow rate: 0.2 mL/min, dual pump, maximum pressure: 70 MPa), a PDA detector was employed. For peptides and linkers decorated with phenylboronic acid derivatives: the separation was conducted on an Aeris Peptide C18 column (50 × 2.1 mm^2^, 3.5 μm) with a gradient elution of 0–60% B in A or 5–70% B in A. The mobile phase consisted of A (0.1% HCOOH in water) and B (0.1% HCOOH in MeCN), with a flow rate of 0.2 mL/min. The elution was carried out at room temperature over a period of either 20 or 15 min. For albumin hydrolyzate, patient’s serum, and milk samples: the separation was conducted on an Aeris Peptide C18 column (100 × 2.1 mm, 1.7 μm) with a gradient elution of 0–50% B in A or 0–60% B in A. The mobile phase consisted of A (0.1% HCOOH in water) and B (0.1% HCOOH in MeCN), with a flow rate of 0.2 mL/min. The elution was carried out at room temperature for a period of 50 min.

### 2.3 UV–Vis Analysis

All UV–vis experiments were conducted on a plate reader: Tecan Infinite M200 Pro, Tecan Group Ltd., Männedorf, Switzerland, in cuvette measurement mode with blanking. The UV–vis analyses were done in the following stages: *(i)* The determination of resin loading: After cleavage from the solid support of an exact weighed amount of selected functionalized resin (10 mg) and lyophilization, the sample was analyzed by UV–vis. The 4-carboxyphenylboronic acid calibration curve, set up at 236 nm, was used to determine the loading of the functionalized TentaGel resin. The sample was, respectively, diluted in 0.1% HCOOH in H_2_O:MeCN (50:50 v/v) and analyzed by UV–vis at a 236 nm wavelength. The calibration curve and the results of the resin loading determination are provided in Figure S6 and Table S2. *(ii)* The determination of concentration of model peptide: the collected samples, after model peptide enrichment using functionalized resin, were analyzed by UV–vis. The Fmoc-Lys(Dabcyl)–OH calibration curve, set up at 455 nm, was used to determine the final concentration of the model peptide after capture. The sample was, respectively, diluted in 0.1% HCOOH in H_2_O:MeCN, (50:50 v/v) and analyzed by UV–vis at a 455 nm wavelength. The calibration curve and the results of the peptide concentration determination are provided in Figure S13 and Table S3.

### 2.4 General Procedure of Synthesis of Functionalized TentaGel Resins

The TentaGel R Ram resin (loading: 0.18 mmol/g) was utilized for the synthesis of functionalized resins according to the Fmoc strategy. All Fmoc derivatives were used in a molar excess of 3 times or appropriately multiplied. The coupling reactions of amino acid residues were carried out using benzotriazol-1-yloxytripyrrolidinophosphonium hexafluorophosphate (PyBOP) (3 equiv) in the presence of N,N-diisopropylethylamine (DIEA) (6 equiv) with ultrasonic agitation developed by Wołczański et al.^47^ The progress of the coupling reaction was monitored with a ninhydrin test. The fluorenylmethyloxycarbonyl protecting group (Fmoc) was removed with 25% piperidine in DMF solution. The 4-methyltrityl protecting group (Mtt) was removed with 1% triisopropylsilane (TIS) in a DCM solution. After the synthesis was completed, the functionalized resin was cleaved from the solid support using standard conditions: TFA/H_2_O/TIS (95:2.5:2.5, v/v/v). The detailed procedures for the synthesis of each resin (Table 1) are provided in Supporting Information, section 2.1.

**Table 1:** List of Synthesized Functionalized Resins

Abbr.	Sequence
TGR1	PhB-Lys(PhB)-TGR
TGR2	4PhB-3Lys-TGR
TGR3	PhB-βAla-Lys(PhB)-TGR
TGR4	PhB-O2Oc-Lys(PhB)-TGR
TGR5	MESNa-CH_2_CO-Lys(PhB)-TGR

### 2.5 General Procedure for Determining the Capture Efficiency of Model Deoxyfructosylated Peptide (**P1**) Capture

The model peptide H–K(Dabcyl)AK(Fru)AF–NH_2_ (**P1**) was used to determine the affinity of the functionalized resins to glycated peptides. The model peptide H–K(Dabcyl)AK(Fru)AF–NH_2_ (**P1**) was manually synthesized on ChemMatrix Rink resin (loading 0.4–0.6 mmol/g) according to the Fmoc protocol with ultrasonic agitation developed by Wołczański et al.^47^ The synthesis was conducted using commercially available amino acid derivatives, PyBOP (3 equiv) as a coupling reagent in the presence of DIEA (6 equiv), and Fmoc-Lys(Boc)(2,3:4,5-di-*O*-isopropylidene-1-deoxyfructopyranosyl)–OH, which was synthesized according to a previously reported procedure.^48^ Once synthesis was complete, the resin underwent washes with DMF (7 × 1 min), DCM (3 × 1 min), THF (3 × 1 min), and Et_2_O (3 × 1 min) and was then dried under vacuum for 3 days at room temperature. The peptide was cleaved from the resin using a TFA/H_2_O/TIS (95:2.5:2.5 v/v/v) mixture for 6 h, precipitated in cold ether, and lyophilized prior to LC–MS analysis. The analytical characteristics of **P1** are provided in Figure S14.

The resin was swollen in ammonium bicarbonate buffer (50 mM, pH = 8, H_2_O:MeCN, 50:50, *v*/*v*) for 30 min. The model peptide (2.5 equiv) was dissolved in the same buffer and added to the resin, which was mixed for 1 h. After this time, the resin was washed with buffer, and unreacted fractions were collected. Next, the cleavage mixture (0.1% HCOOH in H_2_O:MeCN, 50:50, *v*/*v*) was added to the resin and mixed for 1 h. After this time, the resin was washed with the cleavage mixture, and reacted fractions were collected. After lyophilization, the collected fraction was used to determine the capturing efficiency of the **P1** using UV–vis and the prepared Fmoc-Lys(Dabcyl)–OH calibration curve at a 455 nm wavelength. The detailed procedures for the selective enrichment of the sample into glycated peptides, determination of the concentration of the model peptide, and the results are provided in the Supporting Information (section 2.10 and Table S3).

### 2.6 SEM

The measurements were conducted using a Hitachi S-3400N scanning electron microscope equipped with a tungsten cathode. The powder samples were affixed to a double-sided self-adhesive carbon tape. A thin layer of gold was deposited on the surface of each sample by sputtering. Images were recorded for the sputtered parts of the samples using backscattered electrons (BSE) or secondary electrons (SE) at an accelerating voltage of 10 kV. Elemental analysis was performed by using the EDS method in low vacuum mode at a pressure of 30 Pa in the measurement chamber on the unsputtered parts of the samples. EDS measurements were conducted using a Noran System 7 analyzer with a Thermo Scientific UltraDry detector, with a resolution of 129 eV.

## 3 Results and Discussion

**Figure 2:**
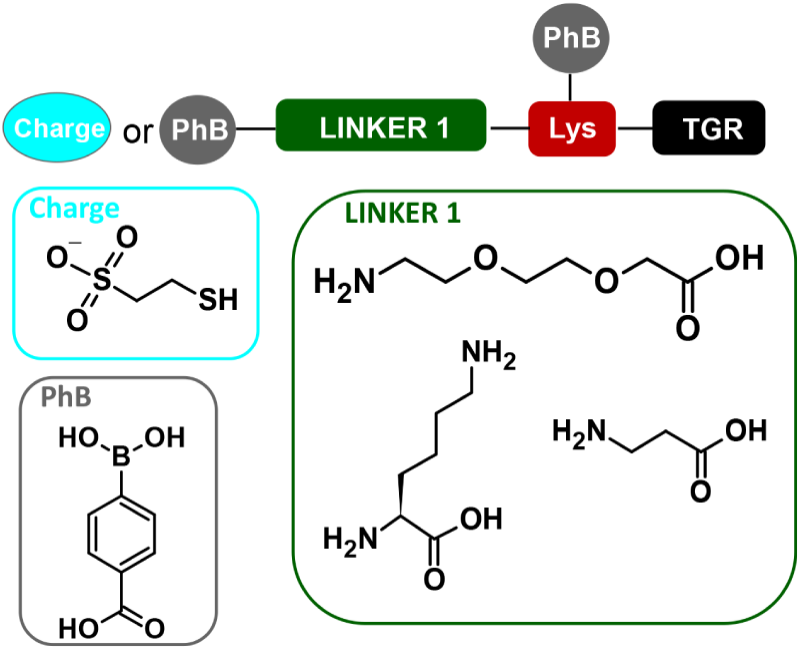
Schematic representation of the structure of functionalized resin.

The paper presents a universal method for the synthesis of any functionalized resin for affinity chromatography, using the example of selectivity to Amadori products due to their significant biological importance. We have chosen the optimal solid support, TentaGel, for the synthesis due to (i) low loading and the possibility to design dendrimeric systems, (ii) low price, and (iii) compatibility with aqueous-organic systems due to the cross-linking of polystyrene with PEG, which is a good alternative to the discontinued ChemMatrix resin. The general synthesis procedure assumes the use of the Fmoc strategy and typical commercially available coupling reagents TBTU, PyBOP, or HATU, according to the coupling procedure in an ultrasonic bath for 15 min.^46,47^ In our protocol, we have demonstrated the universality of the method for the synthesis of several functionalized resins using different linkers, to which the phenylboronic acid moieties were attached, as well as the negative charge group (Figure 2).

Our method involves 5 steps: from the design and synthesis of functionalized resins to the selective capture of glycated peptides from a complex matrix. Importantly, the resins are synthesized without any additional purification steps. Following synthesis completion and loading determination, the functionalized resin is prepared for selective sample enrichment. Moreover, after the capture process, the functionalized resin can be regenerated and reused in a subsequent sample enrichment procedure, although with slightly reduced efficiency.

**Figure 3:**
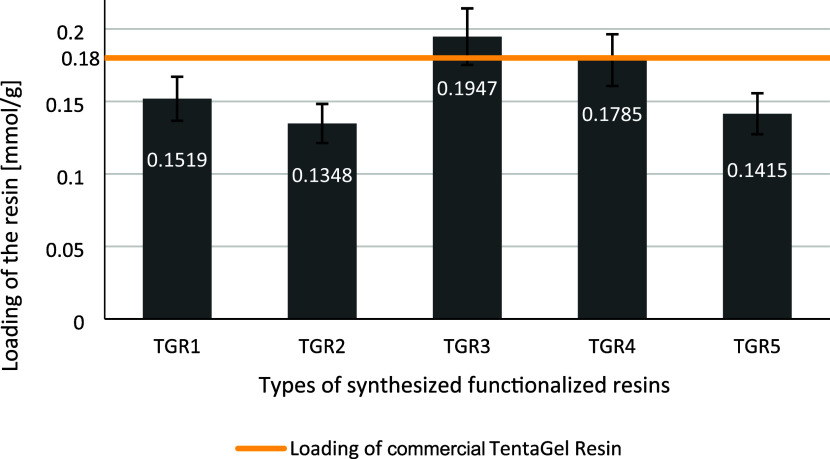
Graphical representation of functionalized resins loading. Loading determined per 2 PhB moiety.

The synthesis (step 1) was conducted by using commercially available Fmoc derivatives, in accordance with the Fmoc strategy for solid-phase synthesis. The detailed synthesis protocols for each resin are provided in the SI, section 2.1.1–2.1.5. Following the synthesis, the next two steps (step 2 and step 3) were to determine the loading and to conduct LC–MS analysis of the linker decorated with PhB removed from TRG (The detailed results and analytical characteristics of functionalized resins are presented in Figures S15–S19). The loading of the modified resin was determined spectrophotometrically. The sample was subjected to cleavage, and the concentration of the liberated product was measured by the UV analysis at 236 nm. The calibration curve was obtained using 4-carboxyphenylboronic acid as a standard (Figure 3). We assumed that at this wavelength, absorption of the product is caused exclusively by the phenyl chromophore. The loading determination was essential to evaluate the maximal capacity of the resin and, subsequently, to determine the efficiency of the resin in binding of glycated/lactosylated peptides. All tests were repeated 3 times, which was taken into account in the measurement error shown on the graph. The amount of product liberated from the resin is in the range 80–110% of the resin loading declared by the manufacturer and depended partially on the efficiency of the synthetic reactions and partially may be attributed to water binding by the TentaGel resin.

**Figure 4:**
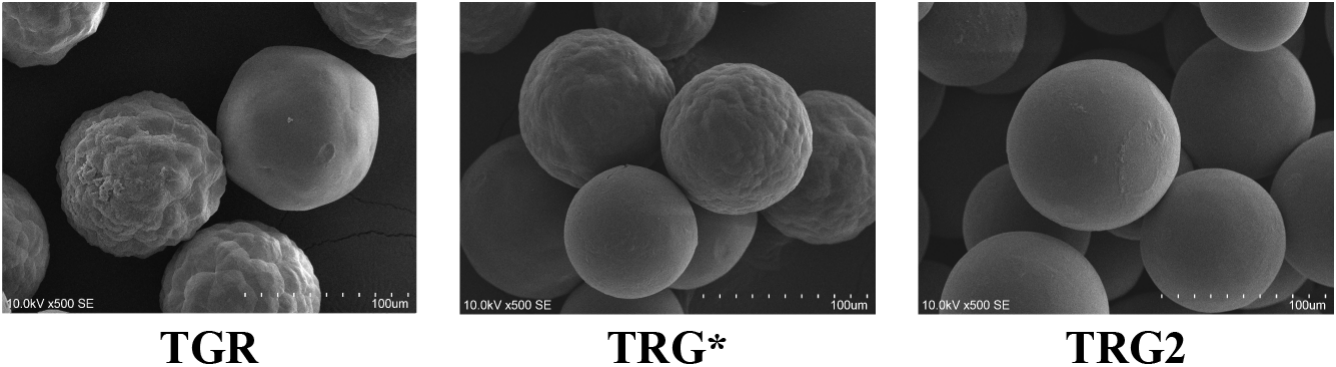
SEM images of the general appearance of the beads. Comparison of the surface morphology of the beads of the TGR, TGR*, and TGR2 samples (TGR: commercially available resin in dry form; TGR*: after swelling in solvents and drying; TGR2: after functionalization and drying).

The surface, shape, and size of the functionalized resin (**TRG2**) beads were analyzed using scanning electron microscopy. Two samples of unmodified resin were also subjected to analysis: **TGR**, a new sample in its dry form, and TGR*, a new sample that had been in contact with solvents used in the synthesis process and subsequently dried under a vacuum. Figure 4 illustrates that the commercial resin sample comprises beads exhibiting a range of surface corrugations, from relatively smooth to markedly corrugated. Following contact with solvents, it can be observed that the solvent occupies the interior of the bead, resulting in a notable reduction in surface irregularities. Following the functionalization of the resin, the surface of all the beads is observed to be smooth, with a few thin and flat islands of irregular shape. Another noticeable effect is an increase in the diameter of the resin beads after chemical modification. The obtained data suggest that the filling of available spaces occurs across the entire surface, with exposure to the external environment. The synthesis of functionalized resin assisted by ultrasound does not result in deformation or local damage to the resin.

**Figure 5:**
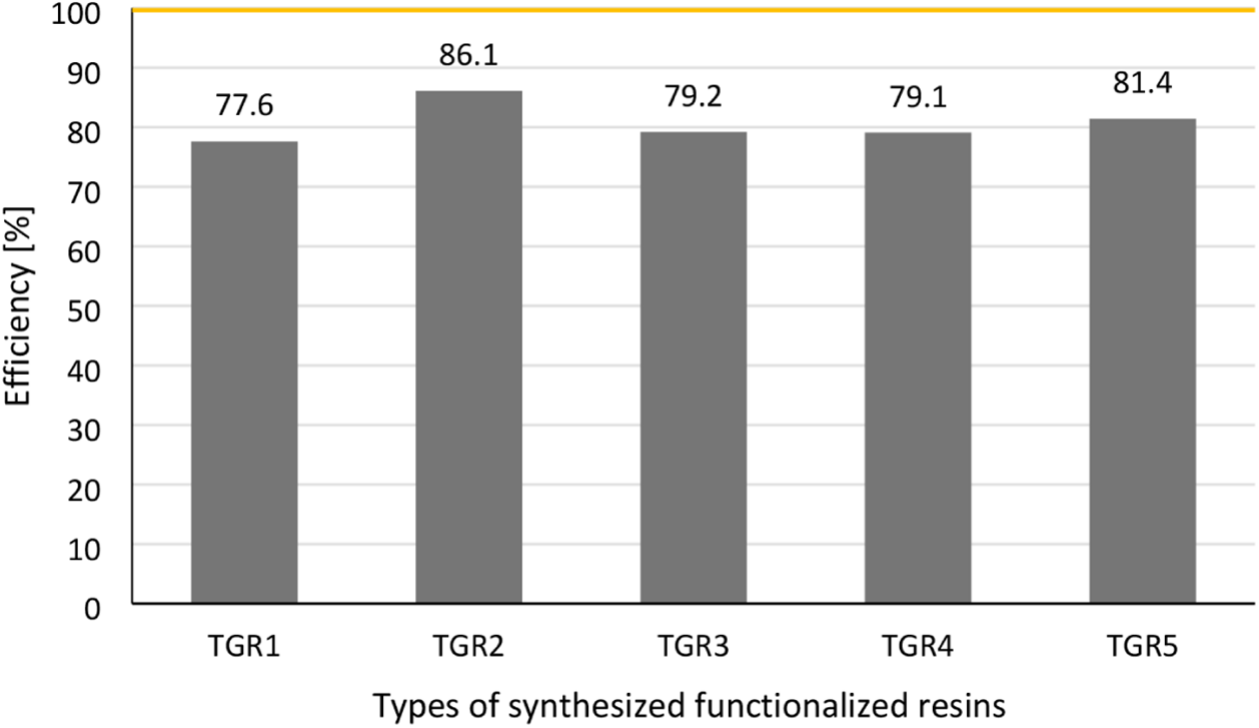
Graphical representation of capture efficiency of model peptides by functionalized resins.

The *fourth step* is to determine the efficiency of the peptide binding by the functionalized resin. In this case, 2.5 equiv of the model peptide **P1** were added to the functionalized resin and subjected to a selective sample enrichment process (see Supporting Information, Section 2.9). The reacted fraction was utilized in UV–vis analysis at 455 nm for the determination of the concentration of **P1** in the sample, thus, allowing the efficiency of the process to be quantified. The obtained results are presented in Figure 5.

Among all of the synthesized resins, 4PhB-3Lys-TGR (**TGR2**) exhibits the highest affinity and efficiency for capturing the model peptide (86%). In our previous study, using the higher-loading resin (ChemMatrix), the use of dendrimeric systems (where the number of PhB units > 4) reduced the capture efficiency. Moreover, the resin could be regenerated and subsequently reused for sample enrichment, albeit with slightly reduced efficiency. Resin regeneration is of limited use in purely analytical work, since the resin is used in small quantities in this application, but it appears to be useful in larger-scale work, such as the preparation of analytical standards. Regeneration consists of a series of washes with ammonium bicarbonate buffer (3 × 1 min), DMF (3 × 1 min), DCM (3 × 1 min), THF (3 × 1 min), and Et_2_O (3 × 1 min). This is followed by drying under vacuum. The resin is then swollen in ammonium bicarbonate buffer and used for subsequent selective enrichment as described in Supporting Information, section 2.9. The regeneration and enrichment process was repeated five times, each time testing the efficiency of the capture process. Up to three repetitions were shown to reduce the efficiency of the process by approximately 10% points. Further repetitions were shown to result in a significant decrease in the process efficiency, rendering the capture of glycated peptides ineffective. Therefore, it is recommended that the resin be used a maximum of three times to realize its full potential for efficient sample enrichment. The collected results are presented in Table S4.

**Figure 6:**
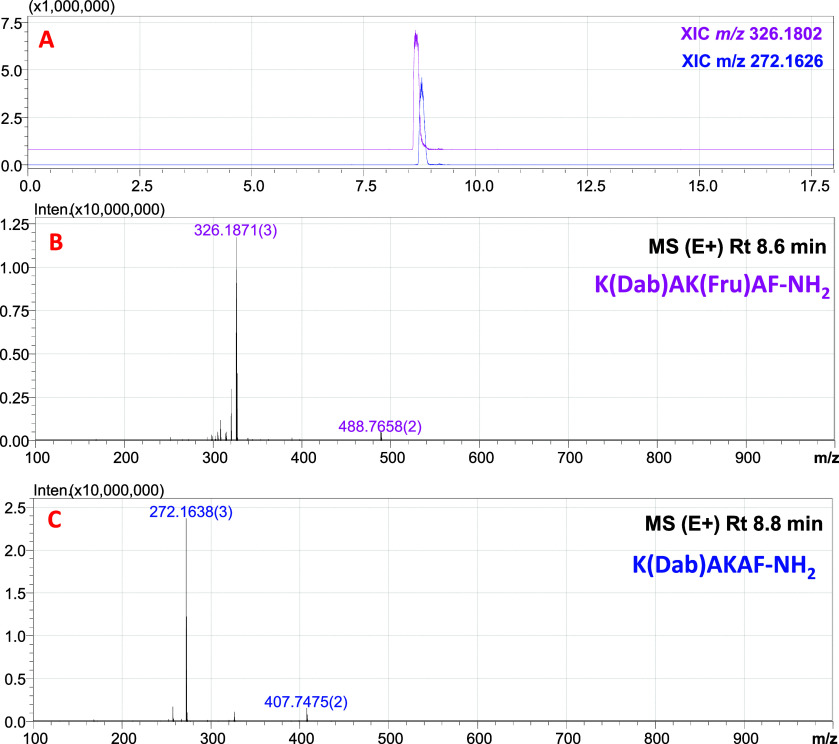
LC–MS analysis of a sample of a mixture of modified and nonmodified model peptide and XIC *m/z* 326.1802 and *m/z* 272.1626 (A); ESI–MS spectrum of the signal with retention time 8.6 min (B); ESI–MS spectrum of the signal with a retention time of 8.8 min (C).

**Figure 7:**
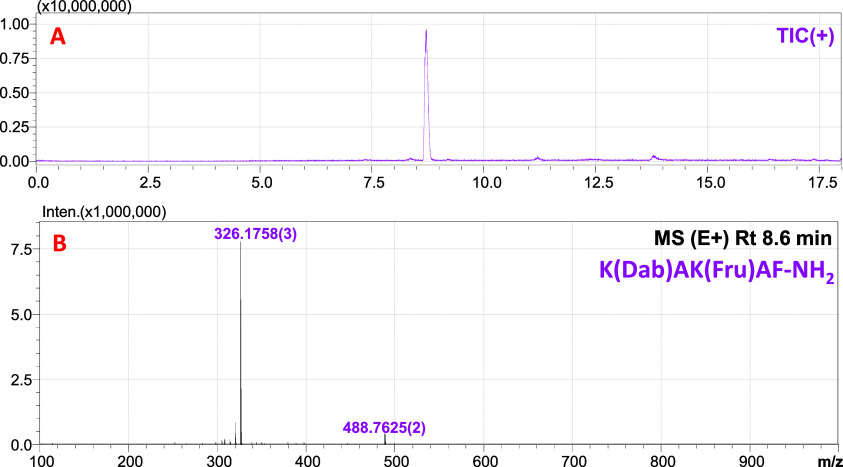
LC–MS analysis of the reacted fraction following the capture of a mixture of modified and nonmodified model peptide by 4PhB-3Lys-TGR (TGR2) (A); ESI–MS spectrum of the signal with retention time 8.6 min (B).

Consequently, resin **TGR2** was selected for subsequent stages of the method. Initially (*the last step*), the resin was tested on a modified (*m*/*z* 326.1817 (2+)) and nonmodified (*m*/*z* 272.1638 (2+)) model peptide system (Figure 6). The LC–MS analysis (Figure 7) of the reacted fraction revealed that resin **TGR2** exhibited a high affinity and selectivity toward glycated peptides. Furthermore, no trace amounts of nonmodified peptide were detected in the reacted fraction.

The **TGR2** resin, which demonstrated the highest efficiency in capturing glycated peptides, was subsequently subjected to further testing on more complex samples. In order to comprehensively assess its potential, samples with a rich matrix were employed to capture the glycated peptides. In the present study, commercially available albumin and a patient’s serum sample were subjected to artificial glycation and hydrolysis. A milk sample was utilized as a biological sample, which was treated with heat, reproducing the standard food preparation. The sample was then subjected to hydrolysis and an Amadori product concentration procedure. The procedures for sample preparation, hydrolysis, and selective enrichment are detailed in the Supporting Information, section 2.4–2.9.

The resin was then tested on a complex matrix of glycated albumin hydrolyzate with or without model peptide **P1** to check how the influence of a rich matrix and competition from other glycated peptides would affect the concentration of model peptide P1. A small quantity (0.00053 mg) of the model peptide was added to the hydrolyzate. The glycated serum albumin was hydrolyzed based on the procedure published by us.^49,50^ The capture process was performed according to the general procedure for selective enrichment of the sample in glycated peptides with functionalized resin (see Supporting Information, section 2.9). The concentration of **P1** was determined by UV–vis basis of calibration curve set up for Fmoc-Lys(Dabcyl)–OH at a wavelength of 455 nm, which demonstrated that **P1** was captured with an efficiency of up to 81%. In spite of a slight reduction in the efficiency, a drop of 5% points, the capture of P1 in the fraction collected from the matrix remained highly efficient. The collected sample was analyzed by the LC-MS/MS method and the PEAKS DB program (see Supporting Information, Section 4). Bioinformatic analysis showed sequence coverage at the level of 67% based on the identification of 77 unique peptide sequences (Figure S23). Although unspecific interaction of nonmodified peptides with the resin was observed (less than 10%), the high sequence coverage from glycated peptides indicates a high degree of specificity. Additionally, the **TGR2** resin was evaluated on a complex authentic sample, the patient’s serum. The blood sample was collected according to the procedure described by Soboleva et al.^51^ Enzymatic hydrolysis was carried out according to the modified procedure introducing the denaturing agent urea.^52^ The details of the procedure are described in the Supporting Information, Section 2.7. A bioinformatics analysis of the patient’s serum hydrolyzate identified a total of 15 different proteins and exhibited 82% sequence coverage of HSA, without any modified glycated peptides. The analysis of the reacted fraction of the sample also exhibited no glycated peptides, thereby precluding the identification of proteins from both the human proteome and albumin (Figures S27, S28, S31, and S32). Accordingly, the patient’s serum was subjected to a glycation reaction in accordance with the procedure previously established for HSA and then subjected to selective enrichment by TGR2. Bioinformatics data from the reacted sample showed 46% sequence coverage of glycated HSA and identified 4 modified proteins. Although nonmodified peptides were present in the sample, 27 out of 34 peptides of HSA were glycated (Figures S29, S30, S33, and S34).

**Figure 8:**
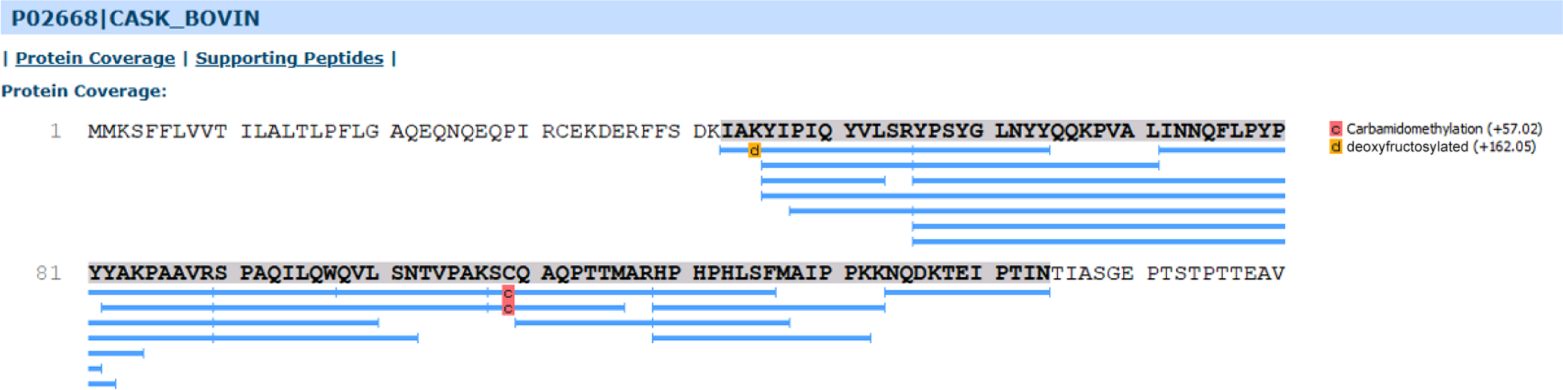
Sequence coverage of κ-casein identified in milk hydrolyzate (reference sample, not heated).

**Figure 9:**
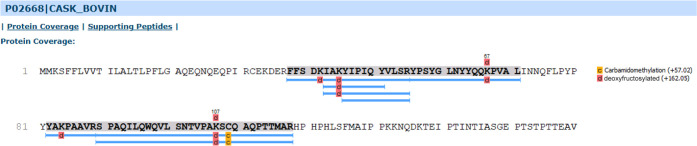
Sequence coverage of κ-casein identified in reacted fraction after selective enrichment of sample by **TGR2**.

The analysis of a milk sample following hydrolysis, as well as a milk sample subjected to both hydrolysis and selective enrichment of glycated peptides, was conducted utilizing the LC–MS/MS and PEAKS DB methods. The analysis of the hydrolyzed samples revealed the presence of various protein sequences. However, it should be noted that, for the purpose of further analysis, only 12 proteins with the highest sequence coverage were considered (Figures 8 and S35). As demonstrated in the case study, lactoglobulins, caseins, immunoglobulins, and albumin were detected with a high percentage coverage. The employment of a functionalized resin (**TGR2**) for the purpose of selective sample enrichment, in conjunction with bioinformatics analysis to identify modified peptides, has enabled the identification of common milk proteins present in the sample. The bioinformatic analysis focused on glycated peptides. The lactoglobulins and caseins present in the sample were identified by analyzing the sequence of the glycated peptides captured (Figure S36). As documented in the literature,^40^ these are the most frequently modified milk proteins. For example, the bioinformatic analysis of kappa-casein showed a sequence coverage of 37%, based on the identification of 9 unique modified peptide sequences (Figure 9). Despite the richness of the matrix and the complexity of the sample, the use of a functionalized resin **TGR2** facilitates the enrichment of the sample with glycated peptides and their subsequent analysis and identification of proteins based on the modified peptide sequence. The resin shows high selectivity for peptide Amadori products, even when used on complex biological samples.

The literature provides several examples of proteomic analyses of milk samples performed to find lysins susceptible to reactions with lactose. Our results are qualitatively consistent with the data published in the literature, indicating the same proteins and lysine residues susceptible to modification. However, a direct comparison of the data obtained here with the literature data is not possible due to the use of a different method of peptide fragmentation (CID in our work, ETD in the work of other authors).^38,40,41^ However, it is clear from the work cited in the literature that the use of ECD and ETD techniques leads to much better sequence coverage and thus allows the identification of a larger number of glycated proteins.^53,54^

The initial phase of the devised methodology entails the design and synthesis of functionalized resin. It is of paramount importance that the resin design be executed with the utmost caution, as the introduction of each additional linker will affect the final outcome. The synthesis of the functionalized resins according to the Fmoc strategy allows for the incorporation of any number or type of linkers. The low loading of the TentaGel resin enables the synthesis of a dendrimeric system with a more complex structure and a higher number of phenylboronic acid moieties (**TGR2** or **TGR5**). The utilization of TentaGel resin and commercially available reagents serves to reduce the cost of the synthesis. Following synthesis, the next stage, cleavage of the crude product from solid support, allows further analysis of the resins. As demonstrated by LC–MS (Figures S15–S19), the functionalized resins are obtained with a high yield and quality, indicating that additional purification is not necessary. The utilization of UV–vis analysis is a fundamental aspect in the determination of resin loading, which is employed in the subsequent step of the determination of the capture efficiency of the model deoxyfructosylated peptide. Once the analytical characterization of the resin has been completed, it is ready to capture Amadori products from a complex sample.

The utilization of a model peptide, comprising a dabcyl moiety and deoxyfructolysine residue, permits the determination of the capture efficiency and affinity of glycated peptides to resins. The addition of a second linker improves the efficiency of peptidyl Amadori product capture, as evidenced by the comparison of resin **TGR3** and **TGR4** with resin **TGR1**. However, the lengthening of the chain of the second linker does not significantly affect the affinity of the resins. The incorporation of a negative charge into the linker (**TGR5**) enhanced the efficiency of model peptide capture, while the selectivity of the process was reduced. This resulted in the resin acting like an ion-exchange resin, thereby exhibiting high affinity not only for glycated peptides but also for positively charged peptides (Figures S20 and S21). The release of the glycated peptide from resin **TGR5** is conditional on the addition of an ionic strength to the cleavage mixture, which in turn necessitates the implementation of an additional step within the overall process. The selected resin **TGR2** was found to exhibit the highest affinity for glycated peptides and was therefore selected for further steps in the method. The efficacy of resin **TGR2** was substantiated through selective enrichment of the glycated peptides derived from a heterogeneous mixture of: (i) modified and nonmodified model peptides; (ii) model peptide within glycated albumin hydrolyzate; (iii) glycated albumin hydrolyzate; (iv) glycated human serum hydrolyzate; and (v) biological sample, i.e., milk. The effective enrichment and analysis of glycated peptides from complex human serum hydrolyzate and milk samples demonstrate the high affinity of the **TGR2** resin for the peptide Amadori product. The obtained results confirm the high coverage of the sequence of albumin in the serum sample and the lactoglobulins and casein in the milk sample, with the use of an excess of the resin, despite nonmodified peptide interaction. It should be noted that unmodified, nonspecifically catching peptides are present in trace amounts compared to the predominant glycated peptides, which is displayed in the PEAKS program (Figures S25 and S26).

While the previously developed ChemMatrix resin yielded a higher percentage of sequence coverage, the degree of protein glycation remains undetermined. To date, the efficiency of sequence coverage by *m*-aminophenylboronic acid-Agarose resin has not been validated. Nevertheless, it has been successfully employed in proteomic studies. The resin has the potential to unambiguously identify proteins and be utilized in the field of proteomic research for both qualitative and quantitative analysis.

## 4 Conclusions

In conclusion, we have developed a universal one-pot method for the synthesis of functionalized resins for the selective enrichment of glycated peptides. The presented method is fast and simple and requires only commercially available reagents. The resins can be modified in various ways, depending on the specific requirements and nature of the sample. The low modification of the TentaGel resin allows the synthesis of a dendrimeric system with four phenylboronic acid moieties, which significantly increases the affinity. The use of the resin for the enrichment of artificially glycated albumin hydrolyzate, patient serum, and milk samples yielded satisfactory results, confirming both the high level of protein sequence coverage and the usefulness of the resin. The improved enrichment efficiency will allow researchers to perform proteomic studies on clinical samples, which are often available in limited amounts, to investigate the role of Amadori products in diabetes, search for new disease biomarkers, and monitor glycated peptide levels in food samples.

Undoubtedly, the main advantage of the proposed approach is the possibility to synthesize other resins modified with functional groups showing affinity to molecules of choice, as well as the rapid and convenient evaluation of the obtained materials. The applied synthetic procedure is based on the well-established and highly efficient protocols used in solid-phase peptide synthesis. The ease of various chemical modifications of PEGs, their chemical stability, and the possibility of immediate material testing allow the design of substrates that capture modified peptides containing not only the *cis*-diol moiety but also carbonyls, sulfhydryls, N-terminal cysteines, histidine-rich sequences, and more.

## Supporting Information Available

LC–MS spectra for all compounds, additional experimental details and methods, bioinformatics data (PDF)
