# Supplementary material for: Affinity on Demand: A One-Pot Method for Synthesis and Sample Enrichment Using TentaGel-Functionalized Resins
Source: ACS Omega. 2025 Apr 22;10(17):18135–44. doi: 10.1021/acsomega.5c02738 (PMC12060058; doi:10.1021/acsomega.5c02738)
Supplement: Supplementary file 1 — ao5c02738_si_001.pdf [file ao5c02738_si_001.pdf]

Supporting Information

Affinity on demand: A One-Pot Method for Synthesis and Sample Enrichment Using TentaGel Functionalized Resins

Michalina Zawadzka[1], Wojciech Gil[1], Andrzej Konieczny[2], Kornelia Krakowska-Jura[2], Monika Kijewska[1]*, and Piotr Stefanowicz[1]*

[1] Faculty of Chemistry, University of Wrocław, Joliot-Curie 14, 50-383 Wrocław, Poland

[2] Department of Nephrology and Transplantation Medicine, Wrocław Medical University, Borowska 213, 50-556 Wrocław, Poland

Corresponding authors*: Monika Kijewska

Faculty of Chemistry, University of Wrocław, F. Joliot-Curie 14, 50-383 Wrocław, Poland, Fax: +48 71 3282348, Tel.: +48-71-3757250

e-mail: monika.kijewska@uwr.edu.pl

Piotr Stefanowicz

Faculty of Chemistry, University of Wrocław, F. Joliot-Curie 14, 50-383 Wrocław, Poland, Fax: +48 71 3282348, Tel.: +48-71-3757213

e-mail: piotr.stefanowicz@uwr.edu.pl

List of contents

1 General Information 3
2 Experimental Section 3
2.1 Synthesis of the functionalized resins 3
2.1.1 PhB-Lys(PhB)-TGR (**TGR1**) 3
2.1.2 4PhB-3Lys-TGR (**TGR2**) 4
2.1.3 PhB-βAla-Lys(PhB)-TGR (**TGR3**) 5
2.1.4 PhB-O2Oc-Lys(PhB)-TGR (**TGR4**) 5
2.1.5 MESNa-CH_2CO-Lys(PhB)-TGR (**TGR5**) 6
2.2 The determination of resin loading 7
2.3 Characterization of TGR2 by SEM method 8
2.3.1 TGR 8
2.3.2 TRG* 10
2.3.3 TRG2 11
2.4 Glycation of human serum albumin 12
2.5 Glycation of patient`s serum sample 13
2.6 Hydrolysis of human serum albumin 13
2.7 Hydrolysis of patient`s serum sample 13
2.8 Milk sample preparation 13
2.9 Selective enrichment of glycated peptides in sample using functionalized resin 13
2.10 The determination of concentration of model peptide 14
3 LC-MS,LC-MS/MS, LC-UV analysis 16
4 Bioinformatics data 23

1 General Information

Bioinformatics analysis

LC-MS/MS analysis of the samples were conducted on Shimadzu qTOF instrument, employing automatic fragmentation mode. The resulting data were the subjected to bioinformatics analysis. The LabSolution software was utilized to convert the .lc data file into mzML format. The data were subsequently subjected to analysis using the PEAKS search engine. To this end, a FASTA file was downloaded from the UniProt database. The following search parameters were established: precursor mass error tolerance ±0.1 Da; fragment ion tolerance ±0.2 Da; collision-induced dissociation (CID) fragmentation, semi-specific digestion mode by trypsin, with up 2 missed cleavages per peptide. Furthermore, carbamidomethylation was selected as a fixed post-translational modification (PTM). Deamidation, dehydration, oxidation of methionine, and hexose modifications were designated as variable post-translational modifications (PTMs), with a maximum of three variable PTMs permitted per peptide. An FDR (false discovery rate) of <2% at the spectra level was employed to filter search results. Furthermore, the presence of at least two unique peptides was established as a criterion for protein identification. The obtained results, particularly the statistical data, have been appended to the Supporting Information.

2 Experimental Section

2.1 Synthesis of the functionalized resins

Table S1 Analytical data for functionalized resin

Abbreviation	Sequence	Found [*m/z*]	Calculated [*m/z*]
**TGR1.1**	PhB-Lys(PhB)-NH_2	442.1959 (1+)	442.1951 (1+)
**TGR2.1**	4PhB-3Lys- NH_2	497.7294 (2+)	497.7296 (2+)
**TGR3.1**	PhB-βAla-Lys(PhB)- NH_2	513.2352 (1+)	513.2322 (1+)
**TGR4.1**	PhB-O2Oc-Lys(PhB)- NH_2	587.2690 (1+)	587.2690 (1+)
**TGR5.1**	MESNa-CH_2CO-Lys(PhB)- NH_2	529.6540 (2-)	529.6523 (2-)

2.1.1 PhB-Lys(PhB)-TGR (**TGR1**)

The TentaGel Resin (loading 0.18 mmol/g) was coupled to Fmoc-Lys(Fmoc)-OH (3 eq) using PyBOP(3 eq) in the presence of DIEA (6 eq) over a 15-minute period under ultrasonic agitation [1]. The reaction was monitored by means of a ninhydrin test. Following the removal of the Fmoc protecting groups by using a 25% piperidine solution in DMF, the 4-carboxyphenylboronic acid (6 eq) was coupled by PyBOP (6 eq) in the presence of DIEA (12 eq) over a period of 2 hours. The functionalized resin was washed with DMF (7x1 min), DCM (3x1 min), THF (3x1 min) and Et2O (3x1 min) and then dried under vacuum at room temperature for three days. PhB-Lys(PhB)-NH_2 (**TGR1.1**) was cleaved from the resin in 2 h using a mixture of TFA/H_2O/TIS (95:2.5:2.5, *v*/*v*/*v*). The solution was evaporated under a gentle nitrogen stream, then lyophilized and used to determine the final loading.

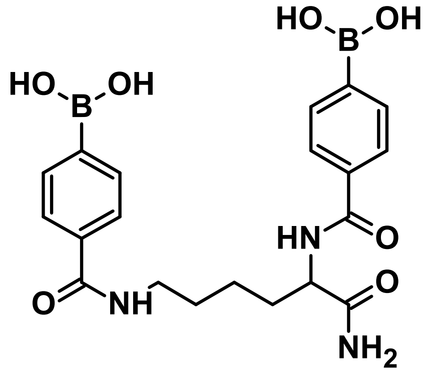

Figure S1 Structural formula of PhB-Lys(PhB)-NH_2 (**TGR1.1**).

2.1.2 4PhB-3Lys-TGR (**TGR2**)

The TentaGel Resin (loading 0.18 mmol/g) was coupled to Fmoc-Lys(Fmoc)-OH (3 eq) using PyBOP(3 eq) in the presence of DIEA (6 eq) over a 15-minute period under ultrasonic agitation [1]. The reaction was monitored by means of a ninhydrin test. Following the removal of the Fmoc protecting groups by using a 25% piperidine solution in DMF, another Fmoc-Lys(Fmoc)-OH (6 eq) residue was coupled using PyBOP (6 eq) in the presence of DIEA (12 eq) for 20 minutes under ultrasonic agitation. Then, 4-carboxyphenylboronic acid (12 eq) was coupled by PyBOP (12 eq) with DIEA (24 eq) in 2h. The functionalized resin was washed with DMF (7x1 min), DCM (3x1 min), THF (3x1 min) and Et_2O (3x1 min) and then dried under vacuum at room temperature for three days. 4PhB-3Lys-NH_2 (**TGR2.1**) was cleaved from the resin in 2 h using a mixture of TFA/H_2O/TIS (95:2.5:2.5, *v*/*v*/*v*). The solution was evaporated under a gentle nitrogen stream, then lyophilized and used to determine the final loading.

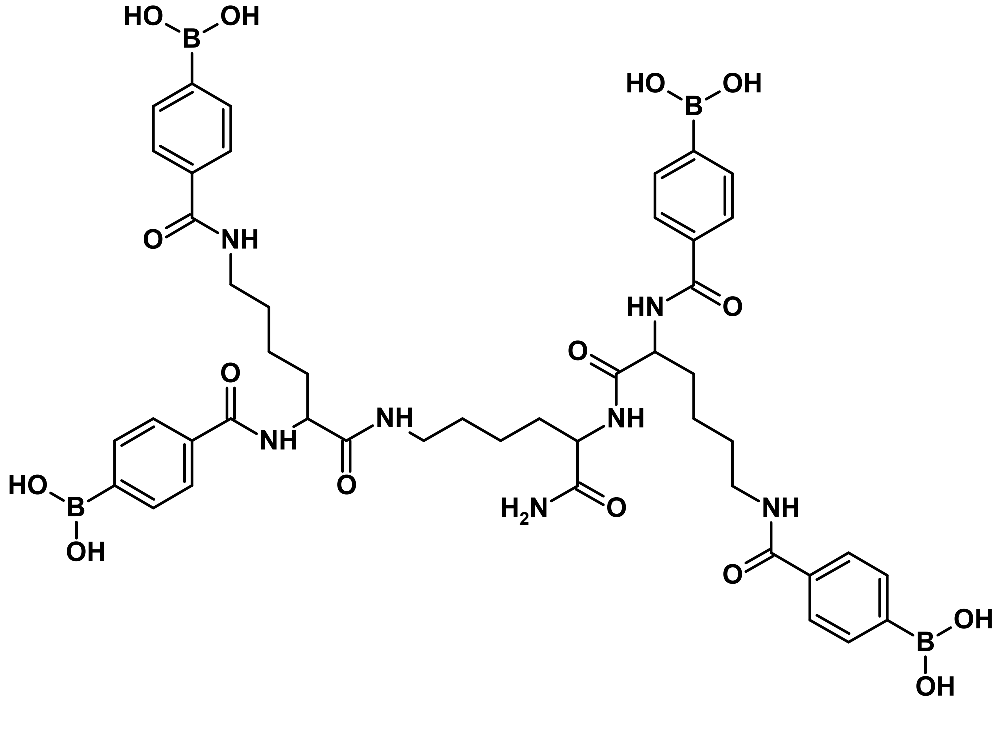

Figure S2 Structural formula of 4PhB-3Lys-NH_2 (**TGR2.1**).

2.1.3 PhB-βAla-Lys(PhB)-TGR (**TGR3**)

The TentaGel Resin (loading 0.18 mmol/g) was coupled to Fmoc-Lys(Mtt)-OH (3 eq) using PyBOP (3 eq) in the presence of DIEA (6 eq) for 15 minutes under ultrasonic agitation [1]. After removal of the Fmoc protecting groups by using a 25% piperidine solution in DMF, the Fmoc-βAla-OH (3 eq) residue was coupled using PyBOP (3 eq) in the presence of DIEA (6 eq) over a period of 15 minutes under ultrasonic agitation. Next, the Mtt protecting group was removed from the ε-amino group of the lysine residue using a 1% solution of TIS in DCM. Then, 4-carboxyphenylboronic acid (12 eq) was coupled by PyBOP (12 eq) with DIEA (24 eq) in 2h. The functionalized resin was washed with DMF (7x1 min), DCM (3x1 min), THF (3x1 min) and Et_2O (3x1 min) and then dried under vacuum at room temperature for three days. PhB-βAla-Lys(PhB)-NH_2 (**TGR3.1**) was cleaved from the resin in 2 h using a mixture of TFA/H_2O/TIS (95:2.5:2.5, *v*/*v*/*v*). The solution was evaporated under a gentle nitrogen stream, then lyophilized and used to determine the final loading.

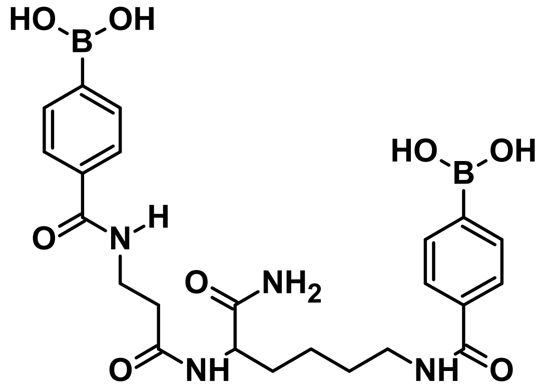

Figure S3 Structural formula of PhB-βAla-Lys(PhB)-NH_2 (**TGR3.1**).

2.1.4 PhB-O2Oc-Lys(PhB)-TGR (**TGR4**)

The TentaGel Resin (loading 0.18 mmol/g) was coupled to Fmoc-Lys(Mtt)-OH (3 eq) using PyBOP (3 eq) in the presence of DIEA (6 eq) for 15 minutes under ultrasonic agitation [1]. After removal of the Fmoc protecting groups by using a 25% piperidine solution in DMF, the Fmoc-O2Oc-OH (3 eq) residue was coupled using PyBOP (3 eq) in the presence of DIEA (6 eq) over a period of 15 minutes under ultrasonic agitation. Next, the Mtt protecting group was removed from the ε-amino group of the lysine residue using a 1% solution of TIS in DCM. Then, 4-carboxyphenylboronic acid (12 eq) was coupled by PyBOP (12 eq) with DIEA (24 eq) in 2h. The functionalized resin was washed with DMF (7x1 min), DCM (3x1 min), THF (3x1 min) and Et_2O (3x1 min) and then dried under vacuum at room temperature for three days. PhB-O2Oc-Lys(PhB)-NH_2 (**TGR4.1**) was cleaved from the resin in 2 h using a mixture of TFA/H_2O/TIS (95:2.5:2.5, *v*/*v*/*v*). The solution was evaporated under a gentle nitrogen stream, then lyophilized and used to determine the final loading.

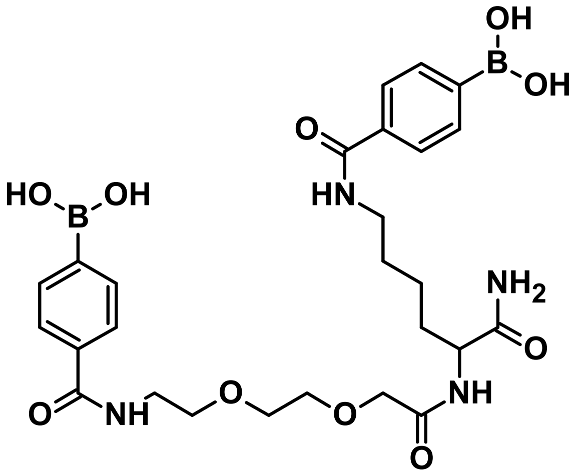

Figure S4 Structural formula PhB-O2Oc-Lys(PhB)-NH_2 (**TGR4.1**).

2.1.5 MESNa-CH_2CO-Lys(PhB)-TGR (**TGR5**)

The TentaGel Resin (loading 0.18 mmol/g) was coupled to Fmoc-Lys(Fmoc)-OH (3 eq) using PyBOP (3 eq) in the presence of DIEA (6 eq) for 15 minutes under ultrasonic agitation [1]. After removal of the Fmoc protecting groups by using a 25% piperidine solution in DMF, the Fmoc-Lys(Mtt)-OH (3 eq) residue was coupled using PyBOP (3 eq) in the presence of DIEA (6 eq) over a period of 15 minutes under ultrasonic agitation. Next, the Mtt protecting group was removed from the ε-amino group of the lysine residue using a 1% solution of TIS in DCM. Then, 4-carboxyphenylboronic acid (12 eq) was coupled by PyBOP (12 eq) with DIEA (24 eq) in 2h. After removal of the Fmoc protecting groups by using a 25% piperidine solution in DMF, bromoacetic acid (6eq) was coupled with DIC (9eq) for 30 minutes. This procedure was repeated three times. The final step involved the MESNa (6 eq) coupling for a 12-hour period. The functionalized resin was washed with DMF (7x1 min), DCM (3x1 min), THF (3x1 min) and Et_2O (3x1 min) and then dried under vacuum at room temperature for three days. MESNa-CH_2CO-Lys(PhB)-NH_2 (**TGR5.1**) was cleaved from the resin in 2 h using a mixture of TFA/H_2O/TIS (95:2.5:2.5, *v*/*v*/*v*). The solution was evaporated under a gentle nitrogen stream, then lyophilized and used to determine the final loading.

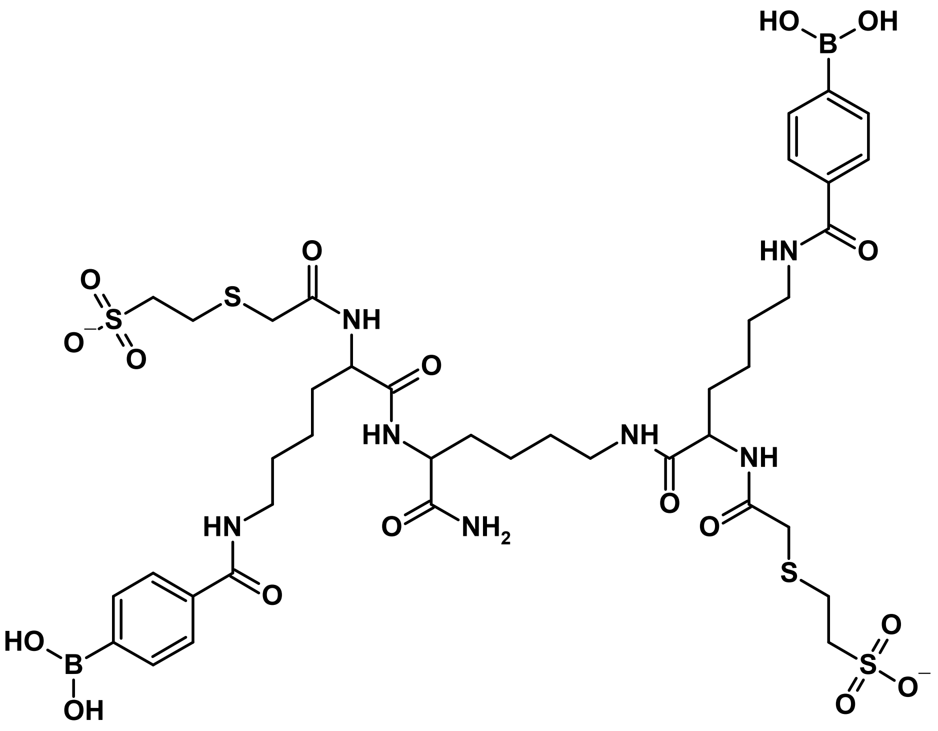

Figure S5 Structural formula MESNa-CH_2CO-Lys(PhB)-NH_2 (**TGR5.1**).

2.2 The determination of resin loading

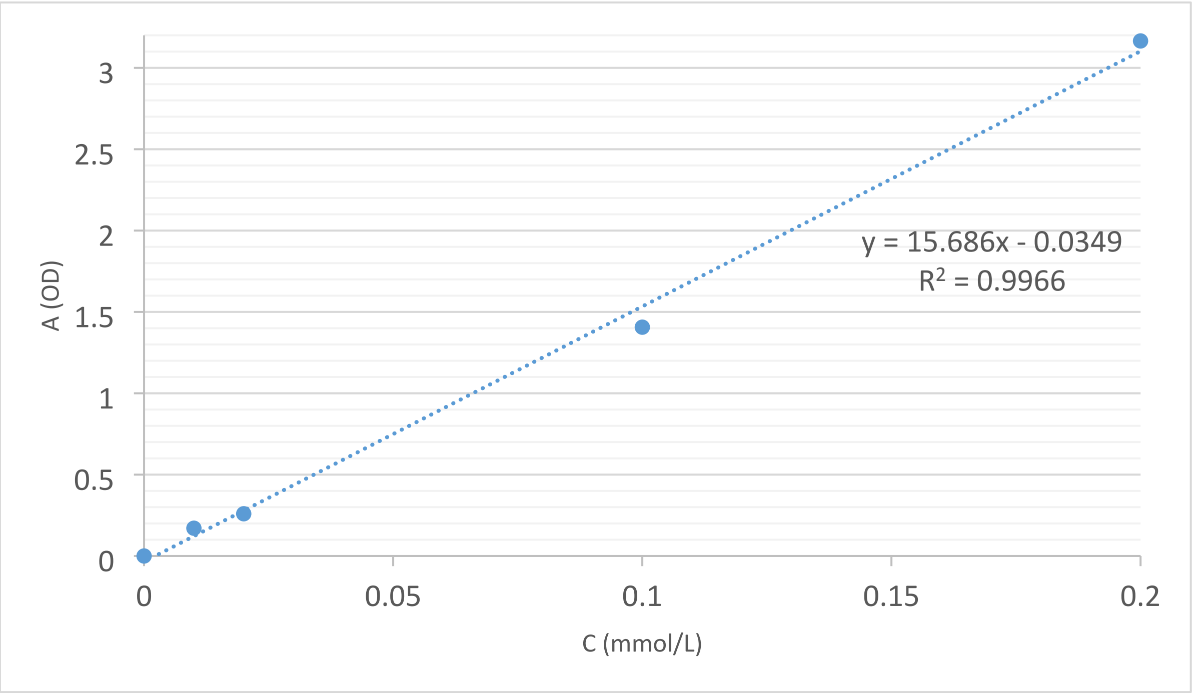

Figure S6 Calibration curve of 4-carboxyphenylboronic acid set up at 236 nm with reported calibration equation.

Table S1 Loading of functionalized resins

Abbrevation	Sequence	mass (g)	Dillution	Abs (OD)	n (mmol)	Loading per 2 PhB moiety (mmol/g)
TGR1	PhB-Lys(PhB)-TGR	0.01	10	1.2295	0.00152	0.1519
TGR2	4PhB-3Lys-TGR	0.01	20	1.0919	0.00270	0.1348
TGR3	PhB-βAla-Lys(PhB)-TGR	0.01	10	1.5925	0.00199	0.1947
TGR4	PhB-O2Oc-Lys(PhB)-TGR	0.01	10	1.3510	0.00168	0.1785
TGR5	MESNa-CH_2CO-Lys(PhB)-TGR	0.01	10	1.1443	0.00141	0.1415

2.3 Characterization of TGR2 by SEM method

Below is a brief description of the SEM microscope used.

Hitachi SEM

• Tungsten cathode gun magnification 80 – 300,000x

• Maximum resolution: 3 nm

• Secondary electron (SE) and backscattered electron (BSE) detector

• Low vacuum secondary electron detector (ESED)

• Thermo Scientific Ultra Dry Lithium Drifted Silicon detector.

• Cressington 108A gold sputtering machine

2.3.1 TGR

The sample consists of spheres with diameters between 50 µm and 110 µm. The surface of the spheres is corrugated to a varying degree, from quite smooth to strongly corrugated (**Figure *S7***). The surface corrugation takes the form of tightly fitted islands with diameters between 12 and 28 µm, with angular edges on the circumference.

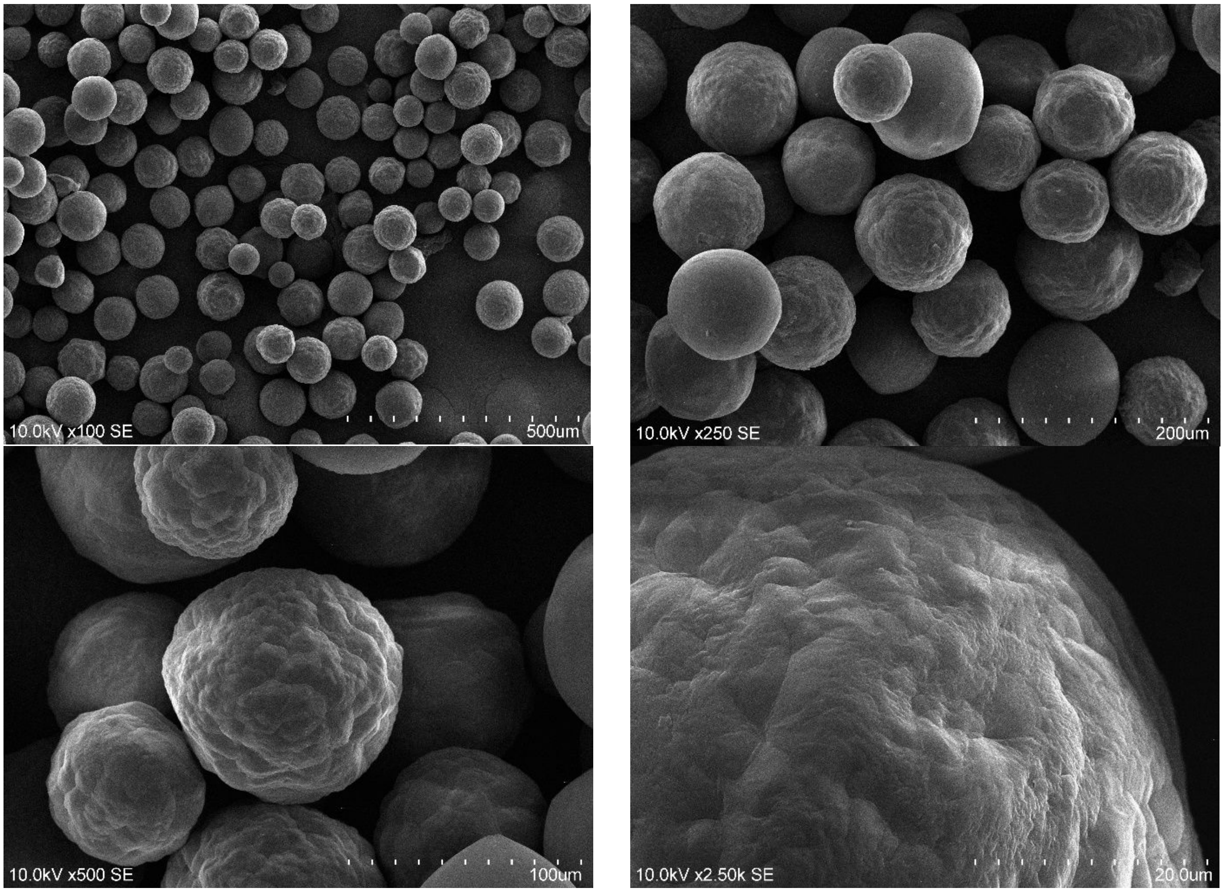

Figure S7 SEM images of the general appearance of beads of the TGR sample (commercially available resin in dry form).

The most numerous spheres are about 100 µm in diameter, the beads size distribution is shown in ***Figure S8***.

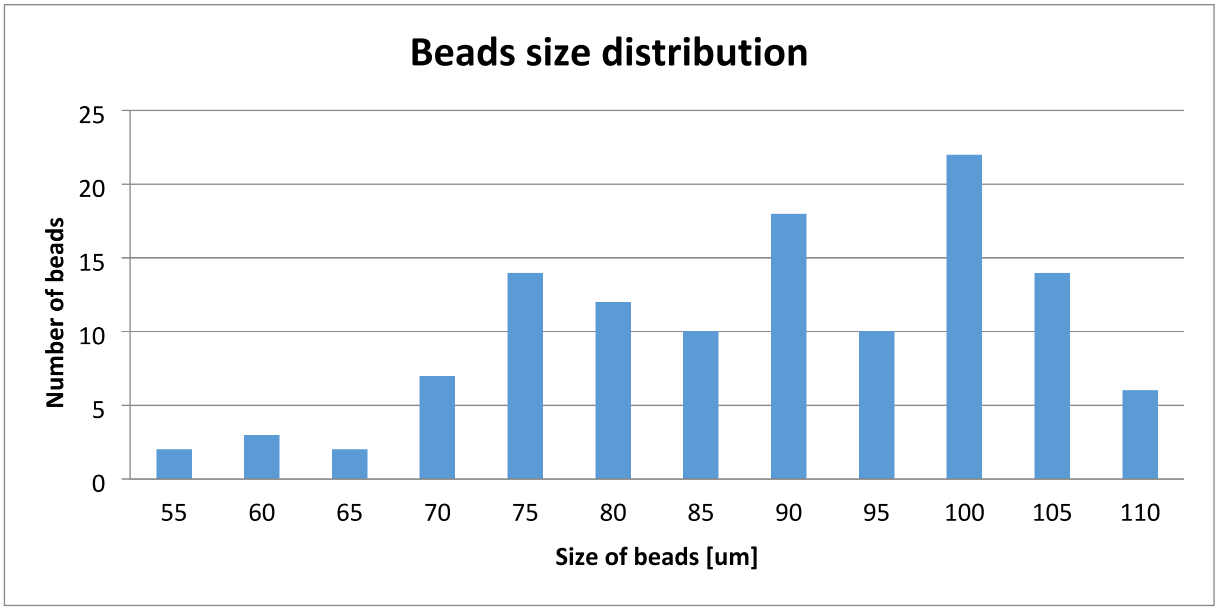

Figure S8 The distribution of beads diameters in the TGR sample.

2.3.2 TRG*

The sample contains round beads with a diameter between 60 μm and 110 μm. The surface of most of the spheres is corrugated similarly to the TGR sample, but a slight decrease in the degree of corrugation is visible. Similarly to the TGR sample, there are also smooth spheres with a few thin and flat islands of irregular shapes (***Figure S9***).

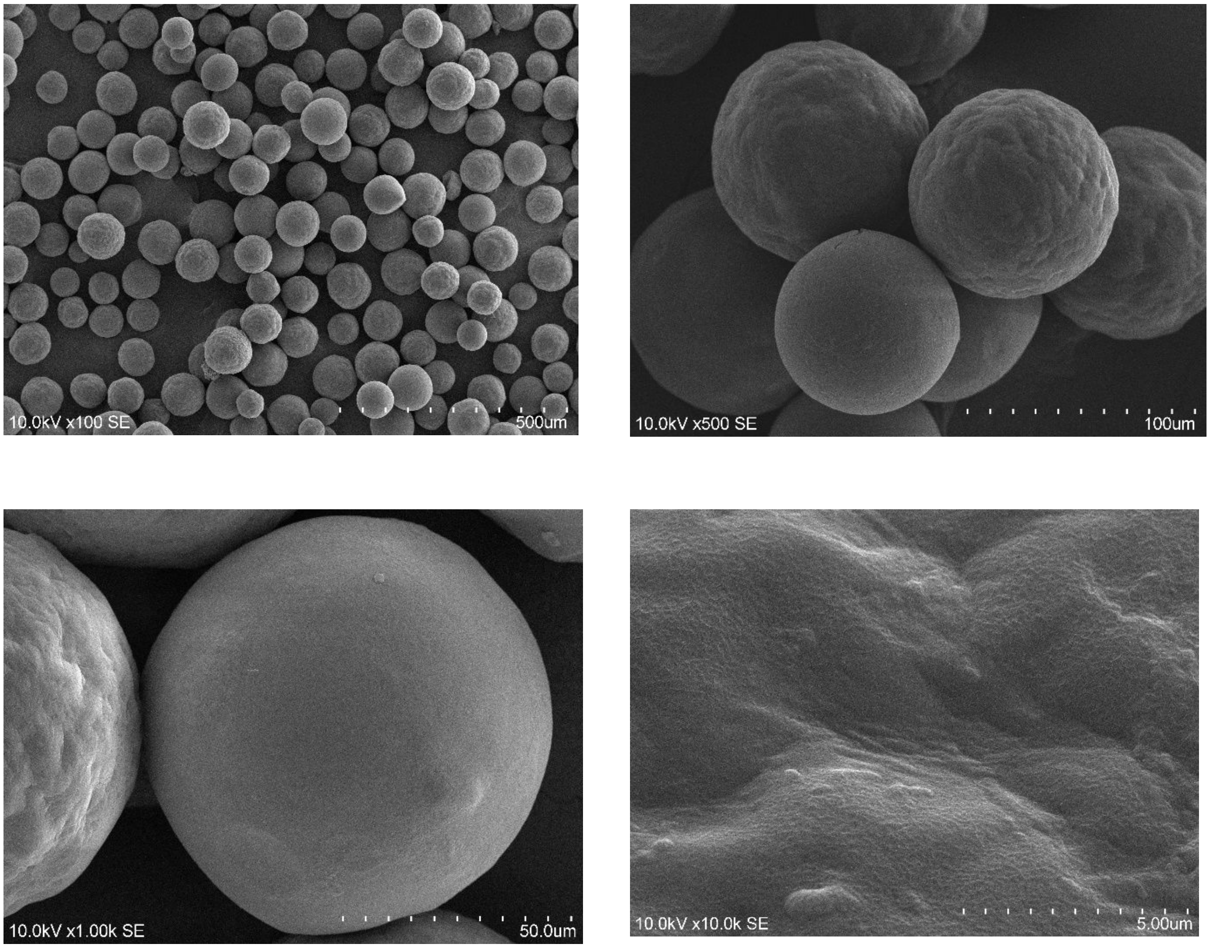

Figure S9 SEM images of the general appearance of beads of the TGR* sample (commercially available resin treated only with solvents used for synthesis and then dried in vacuum).

The sample is predominantly composed of beads with a diameter of approximately 100 μm. The statistical distribution of beads sizes is illustrated in ***Figure S10***.

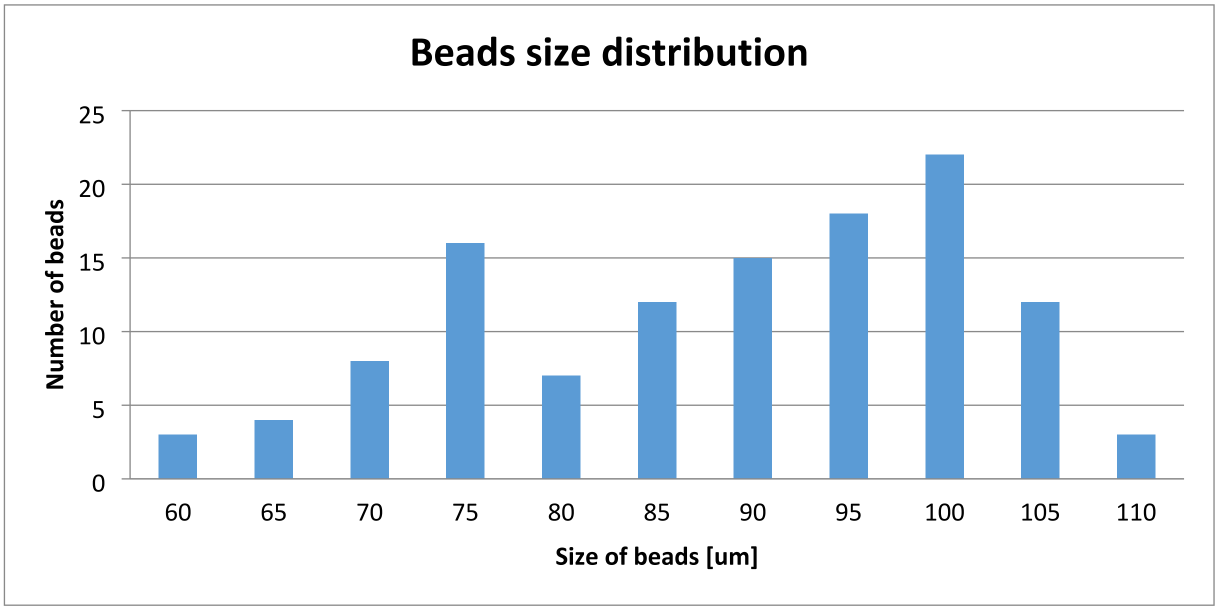

Figure S10 The distribution of beads diameters in the TGR* sample (commercially available resin treated only with solvents used for synthesis and then dried in vacuum).

2.3.3 TRG2

The sample comprises grains with a diameter between 60 µm and 115 µm, characterized by a spherical shape. The surface of all the beads is characterized by a smooth texture, with the presence of a few thin and flat islands of irregular shape (***Figure S11***)

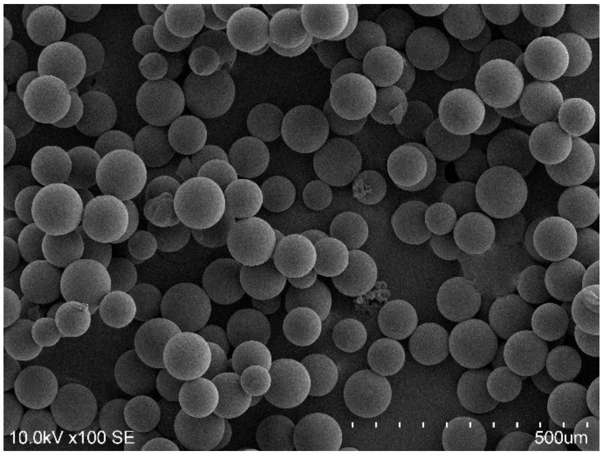

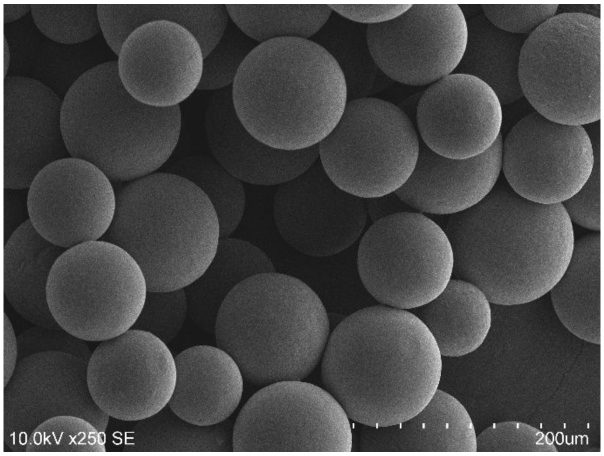

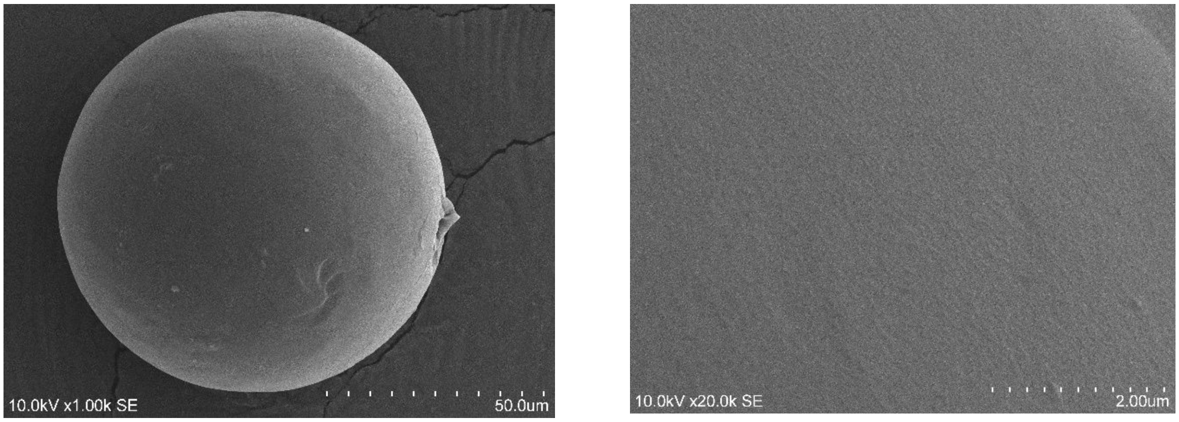

Figure S11 SEM images of the general appearance of the beads of the functionalized resin TGR2 sample (resin loaded with an appropriate linker and phenylboronic acid units, dried in vacuum after synthesis).

The sample is predominantly composed of grains with diameters of approximately 85 μm and 95 μm. The statistical distribution of the bead sizes is illustrated in ***Figure S12***.

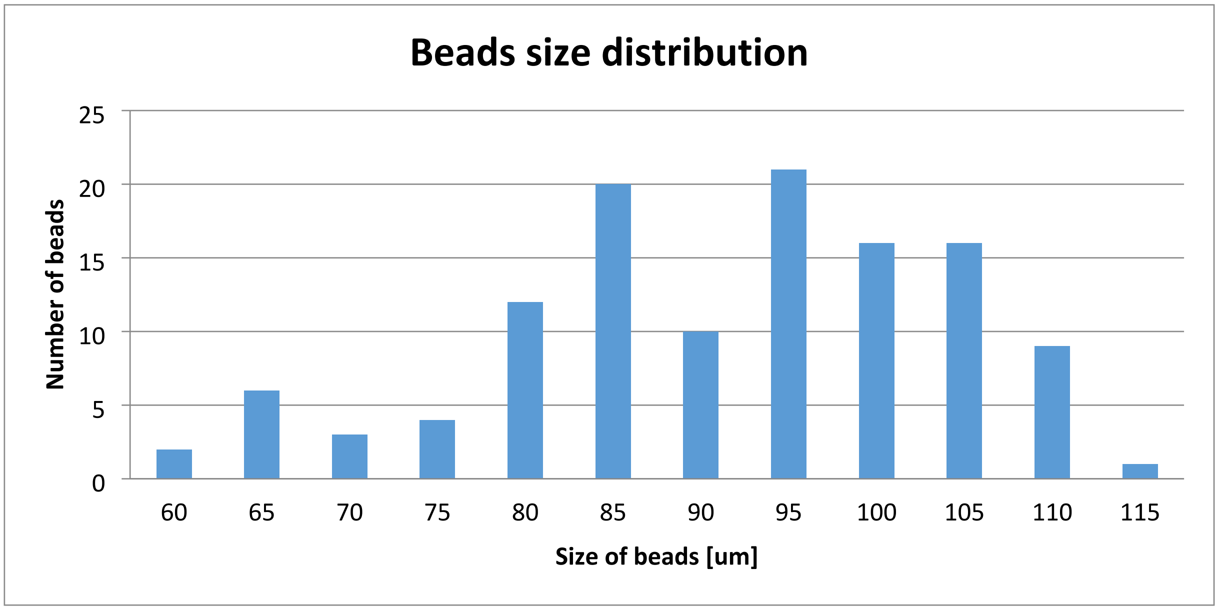

Figure S12 The distribution of beads diameters in the TGR* sample (resin loaded with an appropriate linker and phenylboronic acid units, dried in vacuum after synthesis).

2.4 Glycation of human serum albumin

The solid-phase glycation reaction described by Boratyński and Roy was utilized [2]. Glycation of human serum albumin was carried out in accordance with the our previously published procedure [3,4]. 1 mg of HSA was combined with 1 mg of glucose. The resulting mixture was dissolved in 1 ml of water

and lyophilized. The lyophilized sample was placed in an oven at 80°C for 30 minutes. Following glycation, the sample was subjected to enzymatic hydrolysis, which was catalyzed by trypsin.

2.5 Glycation of patient`s serum sample

The plasma sample aliquots, containing 1 mg of protein, were subjected to the glycation process according to **Chapter 2.4**.

2.6 Hydrolysis of human serum albumin

Following the completion of the glycation process, the sample was subjected to enzymatic hydrolysis in accordance with the methodology previously described [5, 6]. A sample of glycated HSA was dissolved in 200 µl of 50 mM TEAB buffer (pH 8) and 5 µl of a 200 mM DTT solution was added. The mixture was incubated at 60°C for 45 minutes. Subsequently, 4 µl of 1 M iodoacetamide solution was added, and the mixture was incubated for an additional hour. Then, 20 µl of DTT solution and 50 µl of trypsin solution (1mg of trypsin in 1 ml H_2O) were added, and the mixture was incubated at 37°C for 24 hours. After incubation, 5 µl of formic acid was added to quench the reaction, and the sample was lyophilized.

2.7 Hydrolysis of patient`s serum sample

Blood samples (approximately 10 mL each) were collected in polypropylene tubes coated with ethylenediamine tetraacetic acid, EDTA. Afterwards, plasma was separated by centrifugation (1200 g, 15 min) and transferred to 0.5 mL polypropylene tubes [7]. The total plasma protein content was determined by the Bradford assay in a 96-well microtiter plate format as described by Greifenhagen and co-workers [8]. The plasma sample was fractionated into aliquots, each containing 1 mg of protein, which were then subjected to an enzymatic hydrolysis, as detailed in **Chapter 2.6**. Given the presence of diverse proteins within the sample, aliquots were dissolved in an 8 M urea solution in 50 mM TEAB to denaturate the proteins. Prior to the addition of the trypsin solution, the sample was diluted to a final urea concentration of 2 M. This step serves to reactivate the trypsin, thus effecting the completion of the hydrolysis process.

2.8 Milk sample preparation

A quantity of 10 ml of low-fat, lactose-free milk sample was extracted thrice with diethyl ether, and subsequently heated in a water bath at 90°C for a period of one hour. Then, the sample was allowed to cool to room temperature. The milk sample was fractionated into aliquots, each containing 3 mg of protein, which were then subjected to an enzymatic hydrolysis, as detailed in **Chapter 2.6**. As with the patient serum sample, the milk sample was dissolved in the 8 M urea solution in 50 mM TEAB to denature the proteins. As described in **Chapter 2.7**, prior to the incorporation of the trypsin solution, the sample underwent dilution to a concentration of 2M urea. Following hydrolysis, the milk sample was subjected to selective enrichment of glycated peptides by TGR2. The details of this procedure are provided in **Chapter 2.9**.

2.9 Selective enrichment of glycated peptides in sample using functionalized resin

A selected resin (10 mg- for model peptide, 40 mg- for hydrolysate) was swollen in an ammonium bicarbonate buffer (50 mM, pH = 8, H_2O:MeCN, 50:50 *v/v*) for 30 minutes. Thereafter, the model peptide **P1** (2.5 eq) or HSA hydrolysate (with or without P1 0.00053 mg) or patient's serum sample or milk sample was dissolved in 1 ml of the same buffer and added to the syringe, where it was mixed for 1 hour. The mixture was then filtered off, and the resin was washed with buffer three times. All fractions were collected and lyophilized. The cleaved mixture (0.1% HCOOH in H_2O:MeCN, 50:50 *v/v*) was then added to the syringe and mixed for 1h, to remove the reacted glycated peptide. The mixture was filtered off, and the resin was washed with the cleaved mixture three times. The obtained samples were subjected to LC-MS and/or UV-Vis analysis. Following the capture of the model peptide, the resin was washed with ammonium bicarbonate buffer (3x1 min), DMF (3x1 min), DCM (3x1 min), THF (3x1 min), and Et_2O (3x1 min), and subsequently dried under *vacuum*.

2.10 The determination of concentration of model peptide

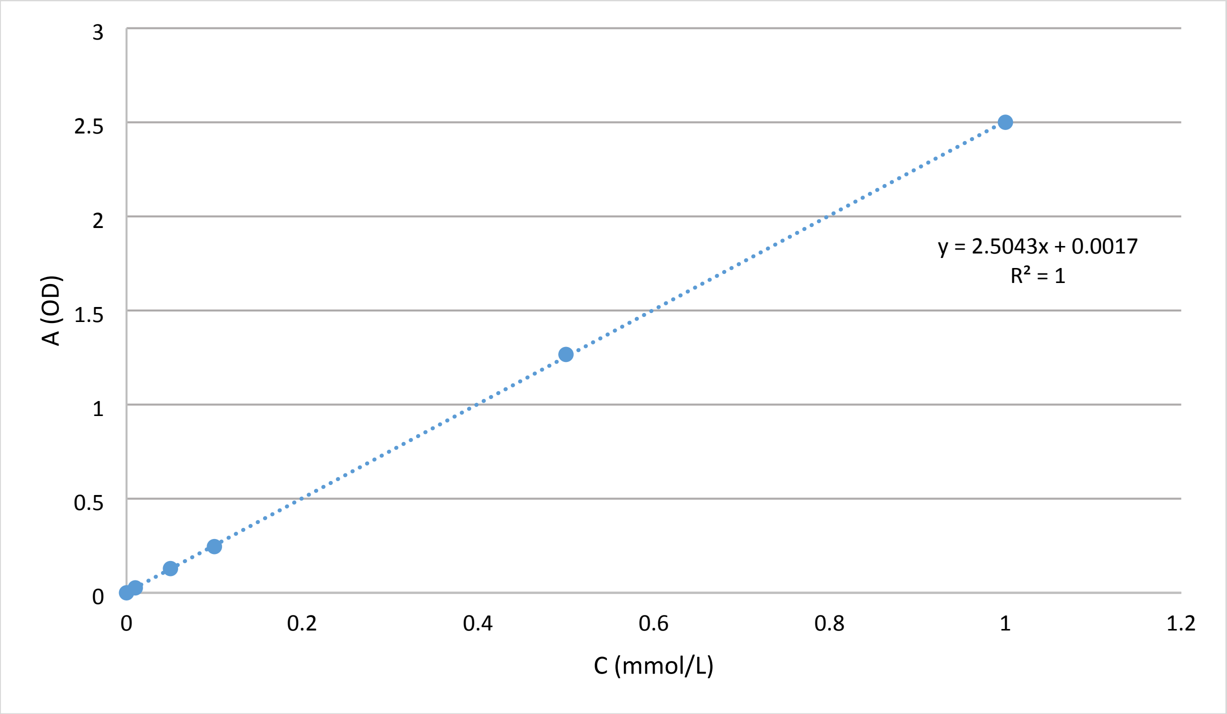

Figure S13 Calibration curve of Fmoc-Lys(Dabcyl)-OH set up at 455 nm with reported calibration equation.

Table S2 The capture efficiency of model peptide by functionalized resins

Abbrevation	Resin	Dillution	Abs (OD)	n (mmol)	Efficiency (%)
**TGR1**	PhB-Lys(PhB)-TGR	10	1.4880	0.00119	77.6
**TGR2**	4PhB-3Lys-TGR	20	1.9372	0.00155	86.1
**TGR3**	PhB-βAla-Lys(PhB)-TGR	10	0.9252	0.00148	79.2
**TGR4**	PhB-O2Oc-Lys(PhB)-TGR	10	1.7797	0.00142	79.1
**TGR5**	MESNa-CH_2CO-Lys(PhB)-TGR	10	1.4449	0.00115	81.4

Table S3 Comparison of capturing efficiency of new and reused batches of **TGR2**

Results	After first use of TGR2	After second use of TGR2	After third use of TGR2	After fourth use of TGR2	After fifth use of TGR2
Abs (OD)	1.9372	1.3947	0.9995	0.8331	0.7051
n (mmol)	0.002225	0.00155	0.000797	0.000712	0.000659
Efficiency (%)	86.1	81.9	74.5	66.8	62.1

Table S4 Glycated albumin hydrolysate doped with H-K(Dabcyl)AK(Fru)AF-NH_2-reacted fraction

Resin	Abs (OD)	n (mmol)	Efficiency (%)
4PhB-3Lys-TGR	0.0586	0.0000045	81.3

3 LC-MS,LC-MS/MS, LC-UV analysis

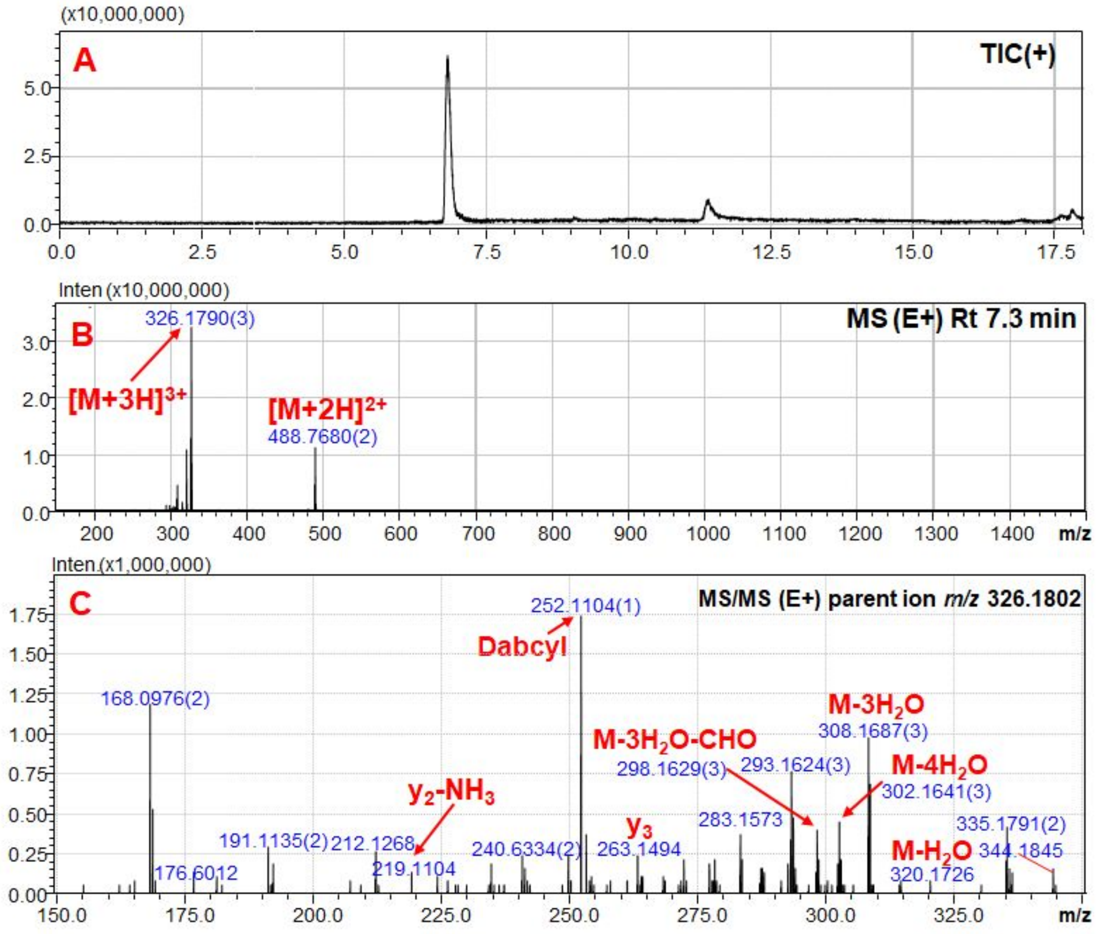

Figure S14 LC-MS chromatogram of H-K(Dabcyl)AK(Fru)AF-NH_2 (**P1**) (A); ESI-MS spectrum of signal with retention time 7.3 min (B); ESI-MS/MS spectrum of signal corresponding to H-K(Dabcyl)AK(Fru)AF-NH_2 (C).

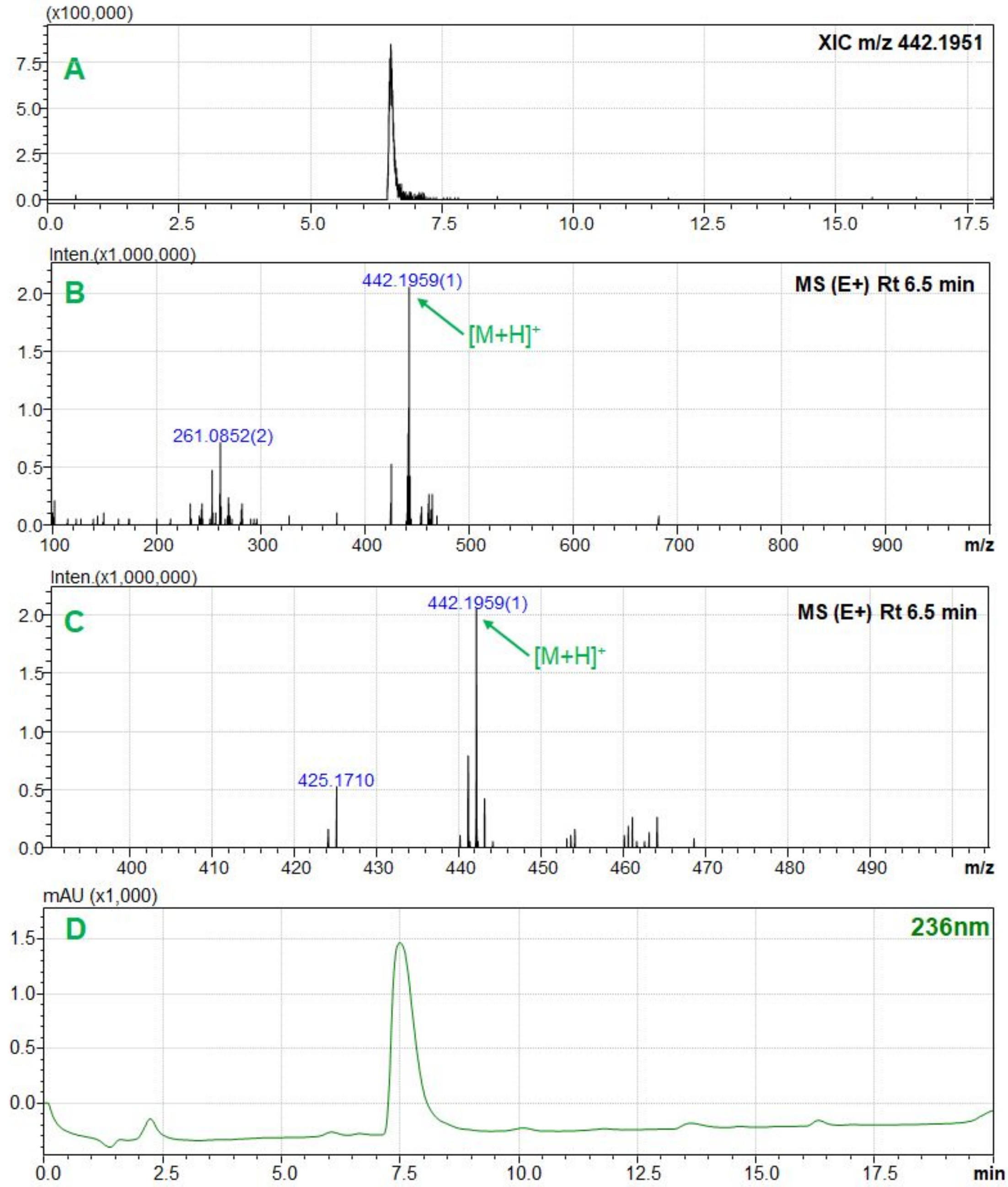

Figure S15 LC-MS analysis of PhB-Lys(PhB)-NH_2 (**TGR1.1**) and XIC *m/z* 442.1959 (*conditions:*0-*60% B in A; The mobile phase consisted of A (0.1% HCOOH in water) and B (0.1% HCOOH in MeCN); flow rate of 0.2 ml/min*) (A); ESI-MS spectrum of signal with retention time 6.5 min (B); ESI-MS spectrum of signal with retention time 6.5 min (range 400-500 *m/z*) (C); LC-UV analysis at wavelength 236 nm (*conditions: 5-70% B in A; The mobile phase consisted of A (0.1% HCOOH in water) and B (0.1% HCOOH in MeCN); flow rate of 0.2 ml/min*) (D).

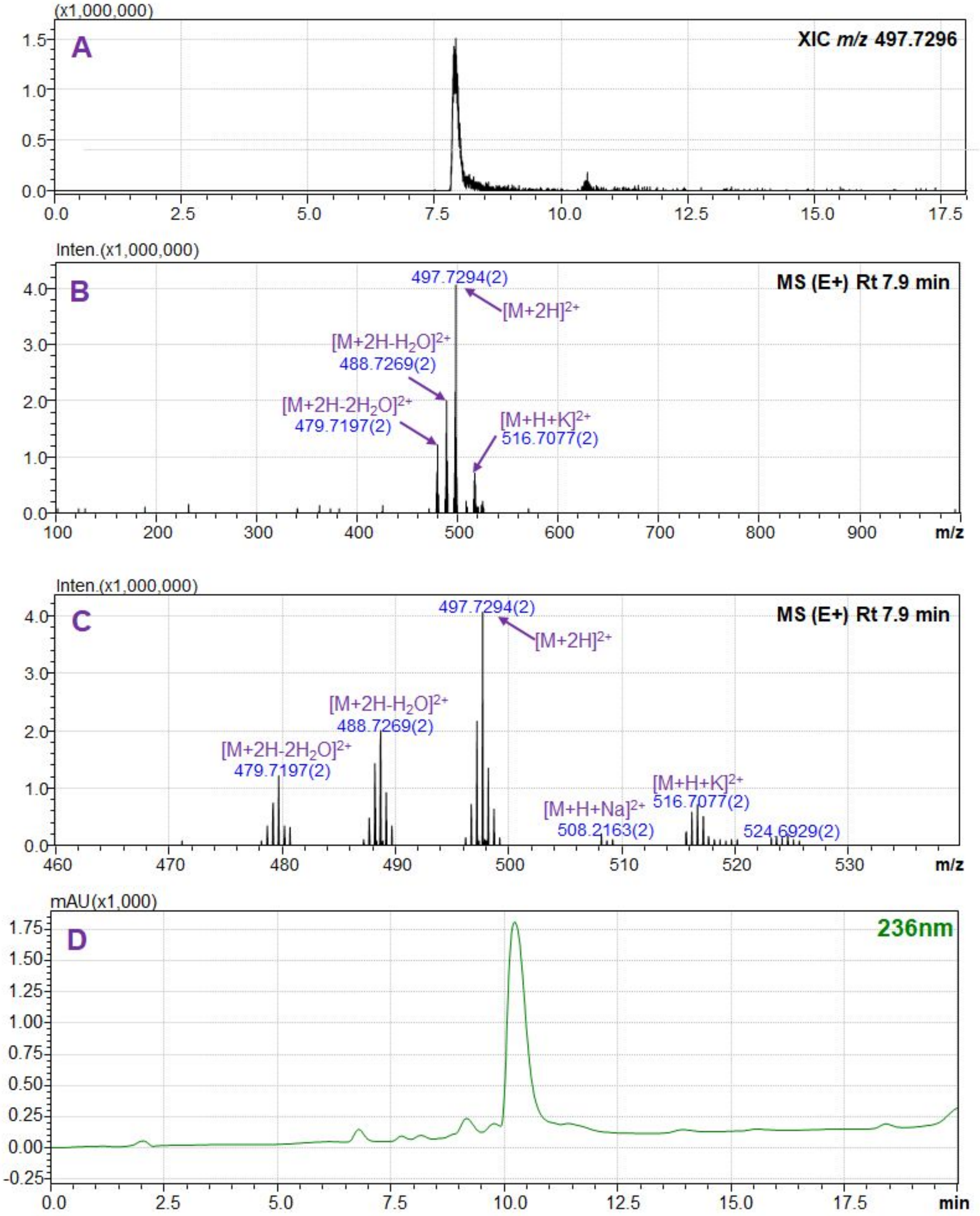

Figure S16 LC-MS analysis of 4PhB-3Lys-NH_2 (**TGR2.1**) and XIC *m/z* 497.7296 (*conditions:0-60% B in A; The mobile phase consisted of A (0.1% HCOOH in water) and B (0.1% HCOOH in MeCN); flow rate of 0.2 ml/min*) (A); ESI-MS spectrum of signal with retention time 7.9 min (B); ESI-MS spectrum of signal with retention time 7.9 min (range 460-540 *m/z*) (C); LC-UV analysis at wavelength 236 nm (*conditions: 5-70% B in A; The mobile phase consisted of A (0.1% HCOOH in water) and B (0.1% HCOOH in MeCN); flow rate of 0.2 ml/min*) (D).

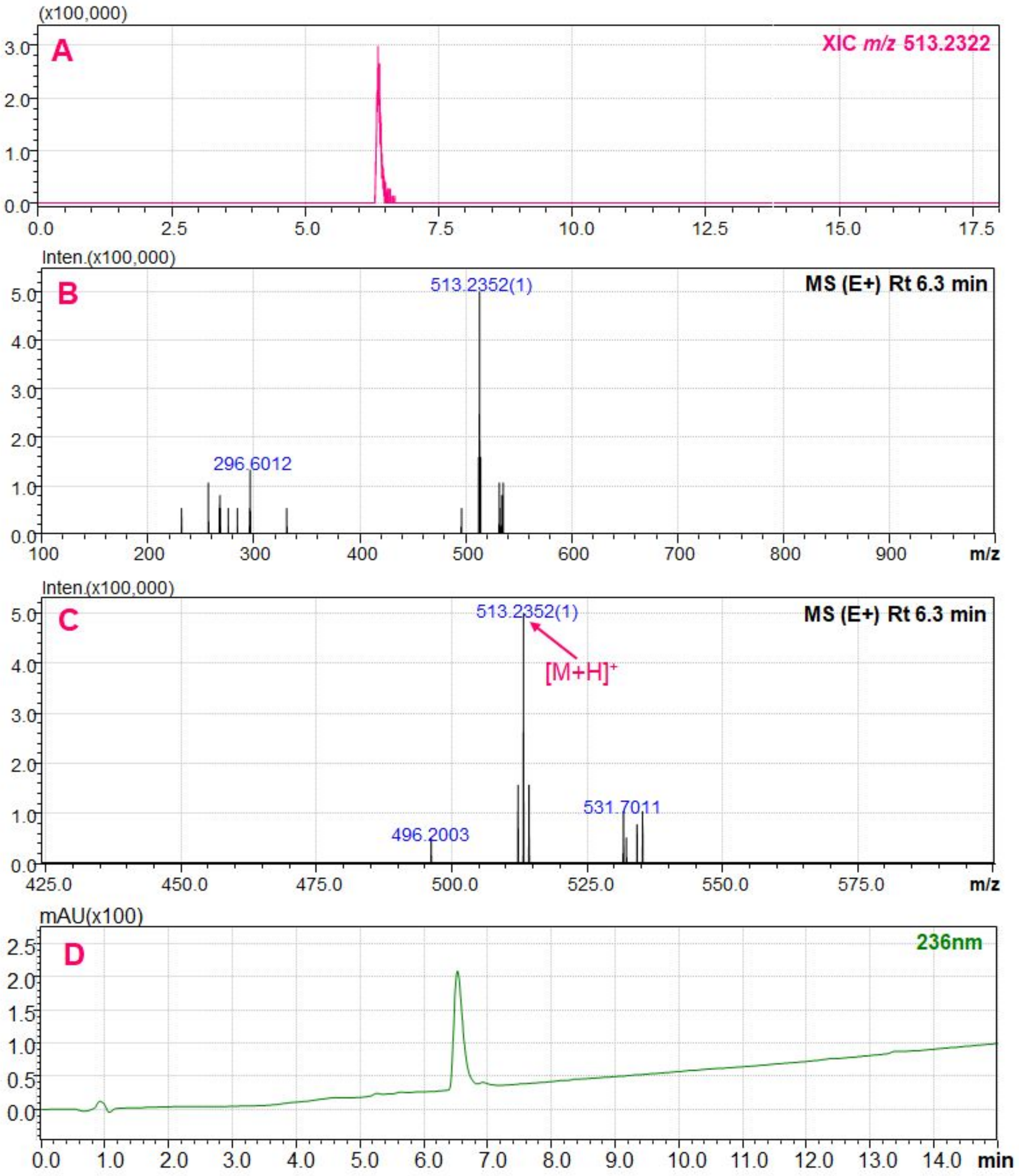

Figure S17 LC-MS analysis of PhB-βAla-Lys(PhB)-NH_2 (**TGR3.1**) and XIC *m/z* 513.2322 (A); ESI-MS spectrum of signal with retention time 6.3 min (B); ESI-MS spectrum of signal with retention time 6.3 min (range 425-590 *m/z*) (C); LC-UV analysis at wavelength 236 nm (D) (*conditions:0-60% B in A; The mobile phase consisted of A (0.1% HCOOH in water) and B (0.1% HCOOH in MeCN); flow rate of 0.2 ml/min*).

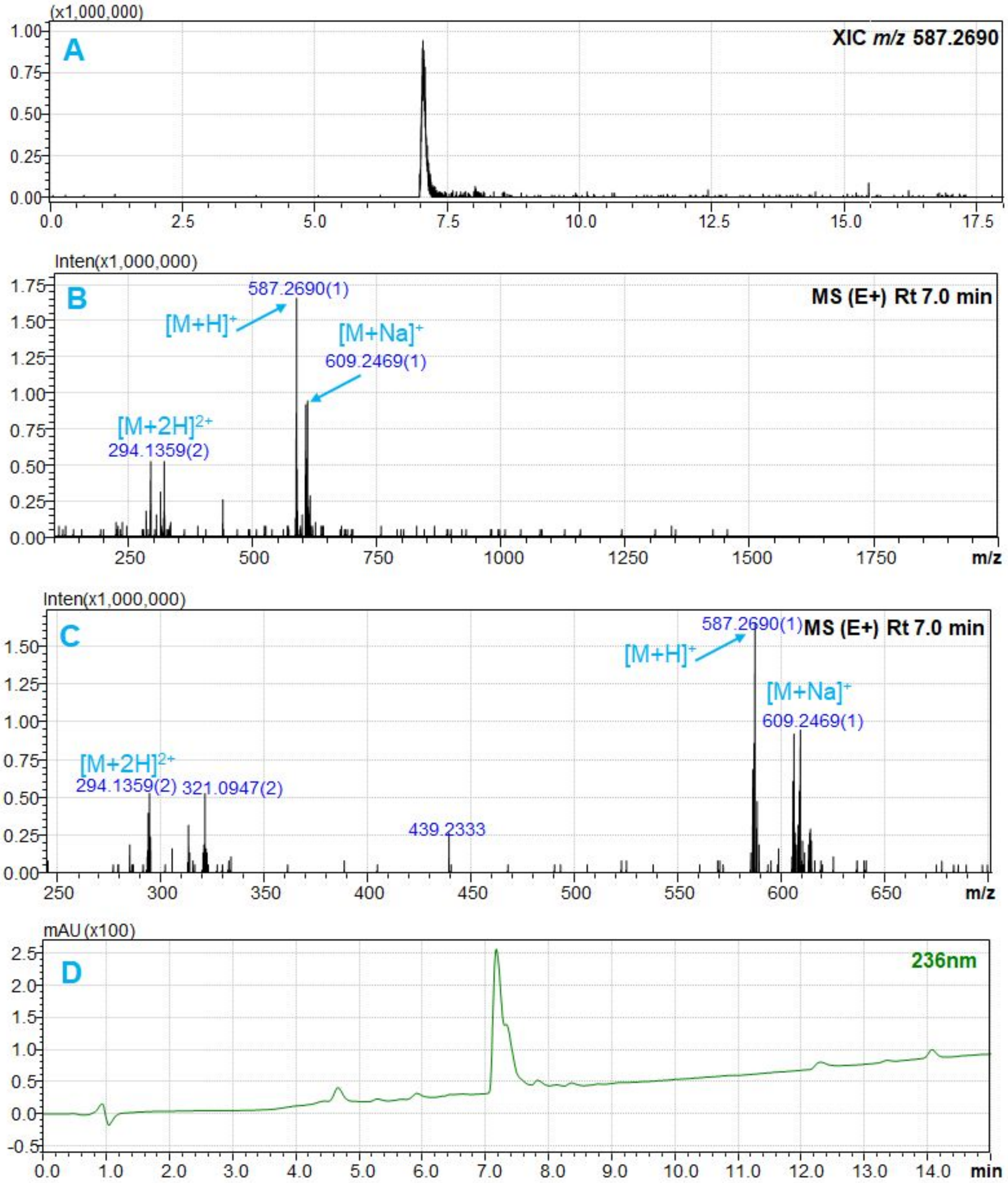

Figure S18 LC-MS analysis of PhB-O2Oc-Lys(PhB)-NH_2 (**TGR4.1**) and XIC *m/z* 587.2690 (A); ESI-MS spectrum of signal with retention time 7.0 min (B); ESI-MS spectrum of signal with retention time 7.0 min (range 250-650 *m/z*) (C); LC-UV analysis at wavelength 236 nm (D) (*conditions:0-60% B in A; The mobile phase consisted of A (0.1% HCOOH in water) and B (0.1% HCOOH in MeCN); flow rate of 0.2 ml/min*).

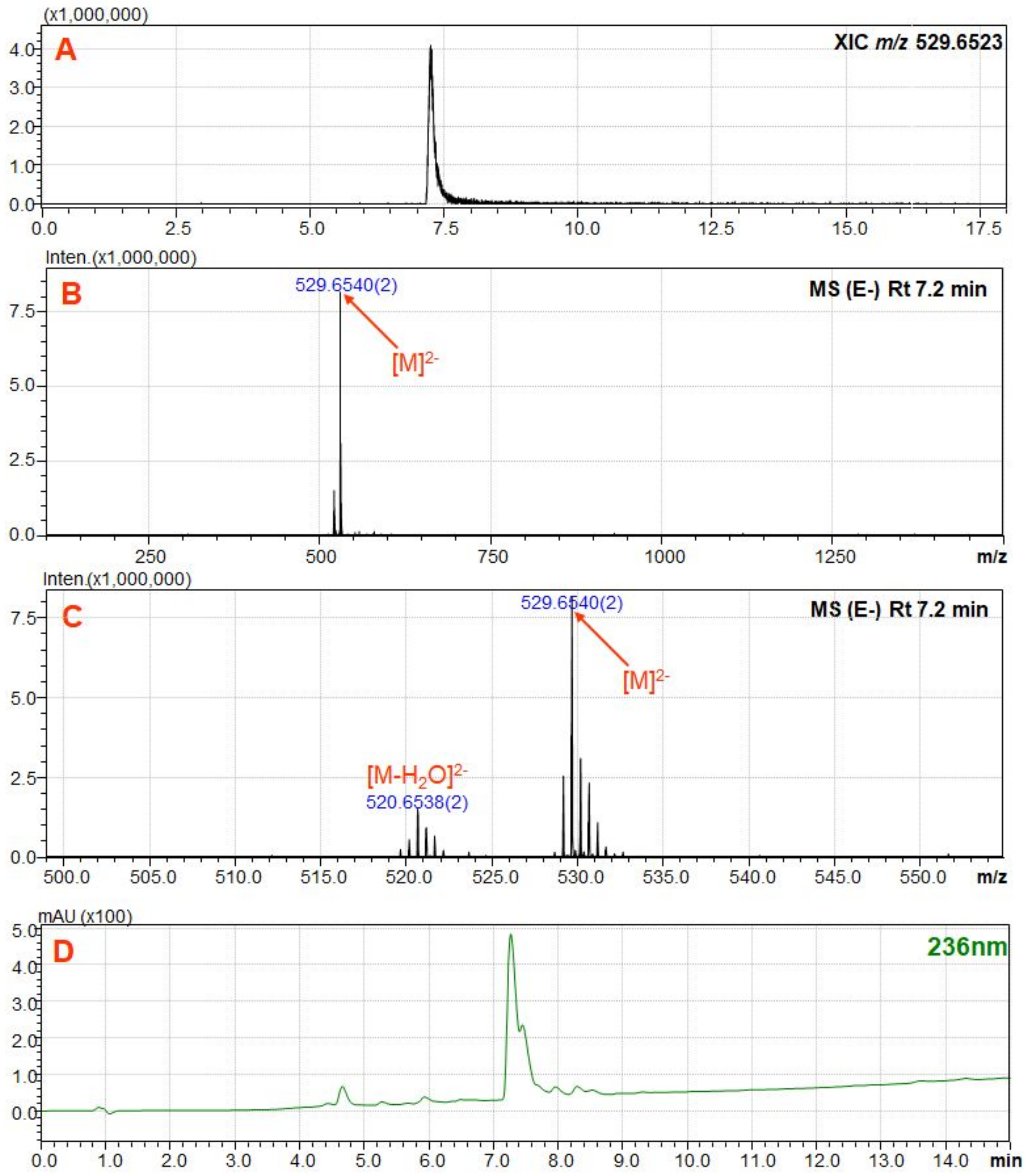

Figure S19 LC-MS analysis of MESNa-CH_2CO-Lys(PhB)-NH_2 (**TGR5.1**) and XIC *m/z* 529.6523 (A); ESI-MS spectrum of signal with retention time 7.2 min (B); ESI-MS spectrum of signal with retention time 7.2 min (range 500-550 *m/z*) (C); LC-UV analysis at wavelength 236 nm (D) (*conditions:0-60% B in A; The mobile phase consisted of A (0.1% HCOOH in water) and B (0.1% HCOOH in MeCN); flow rate of 0.2 ml/min*).

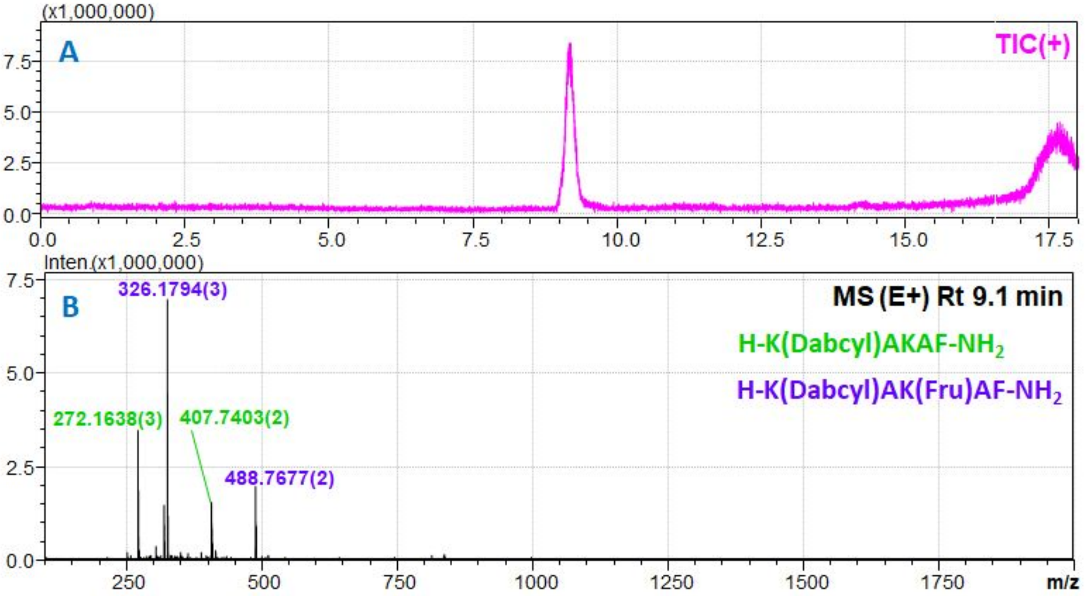

Figure S20 LC-MS analysis of the reacted fraction following the capture of the model peptide by **TGR5** (A); ESI-MS spectrum of the signal with retention time 9.1 min (B) (*conditions:0-60% B in A; The mobile phase consisted of A (0.1% HCOOH in water) and B (0.1% HCOOH in MeCN); flow rate of 0.2 ml/min*).

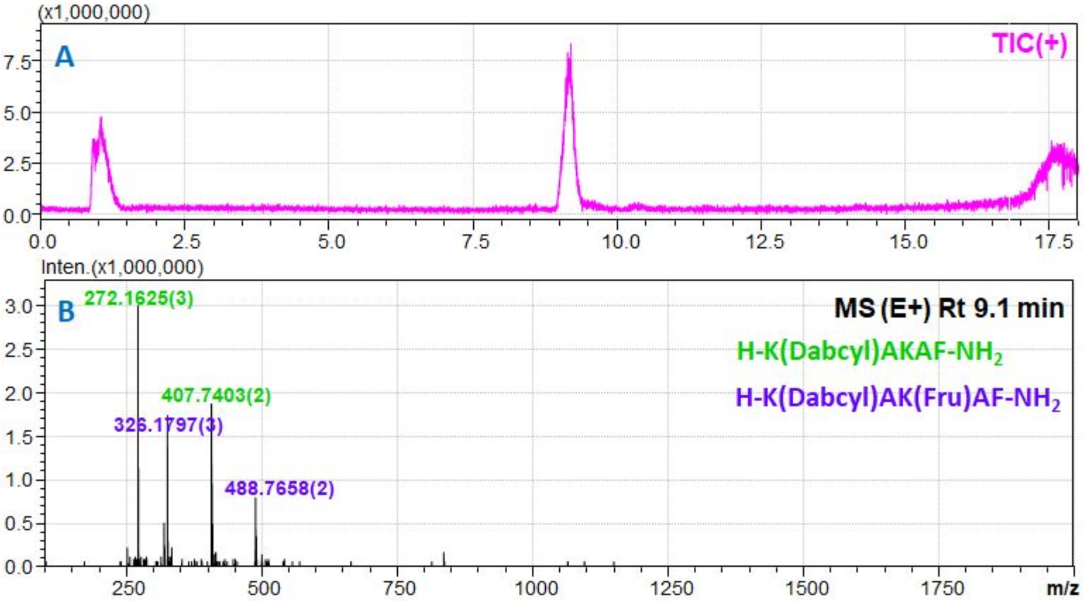

Figure S21 LC-MS analysis of the reacted fraction with addition of ionic strength in cleavage mixture following the capture of the model peptide by **TGR5** (A); ESI-MS spectrum of the signal with retention time 9.1 min (B) (*conditions:0-60% B in A; The mobile phase consisted of A (0.1% HCOOH in water) and B (0.1% HCOOH in MeCN); flow rate of 0.2 ml/min*).

4 Bioinformatics data

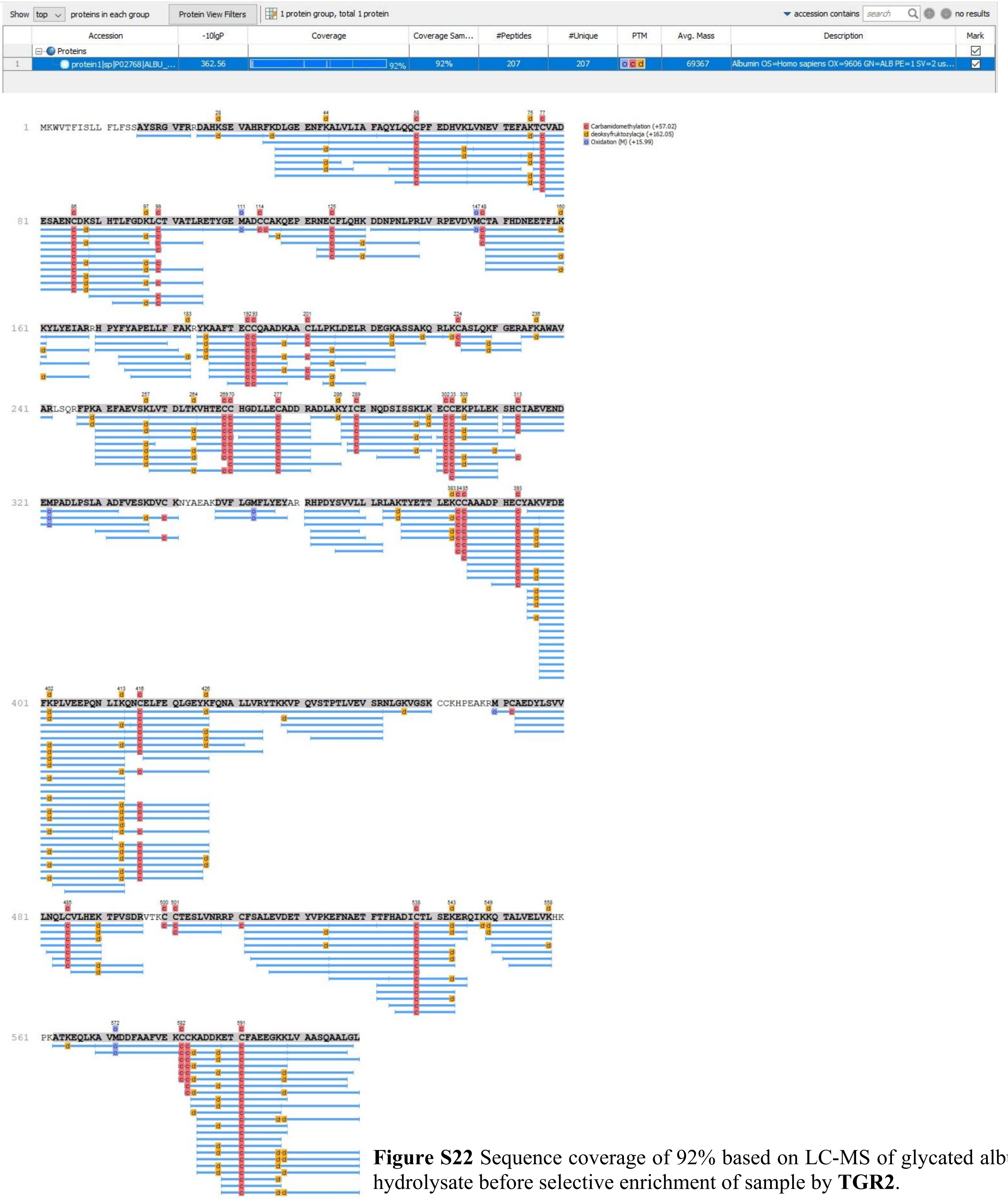

Figure S22 Sequence coverage of 92% based on LC-MS of glycated albumin hydrolysate before selective enrichment of sample by **TGR2**.

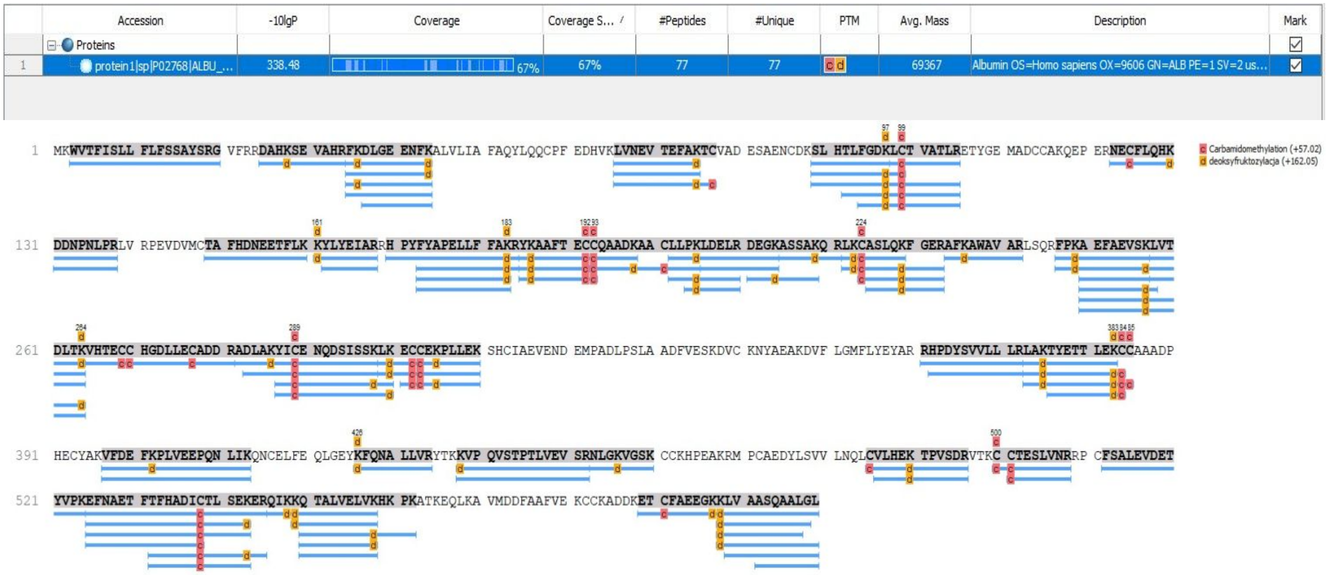

Figure S23 Sequence coverage of 67% based on LC-MS of glycated albumin hydrolysate after selective enrichment of sample by **TGR2**.

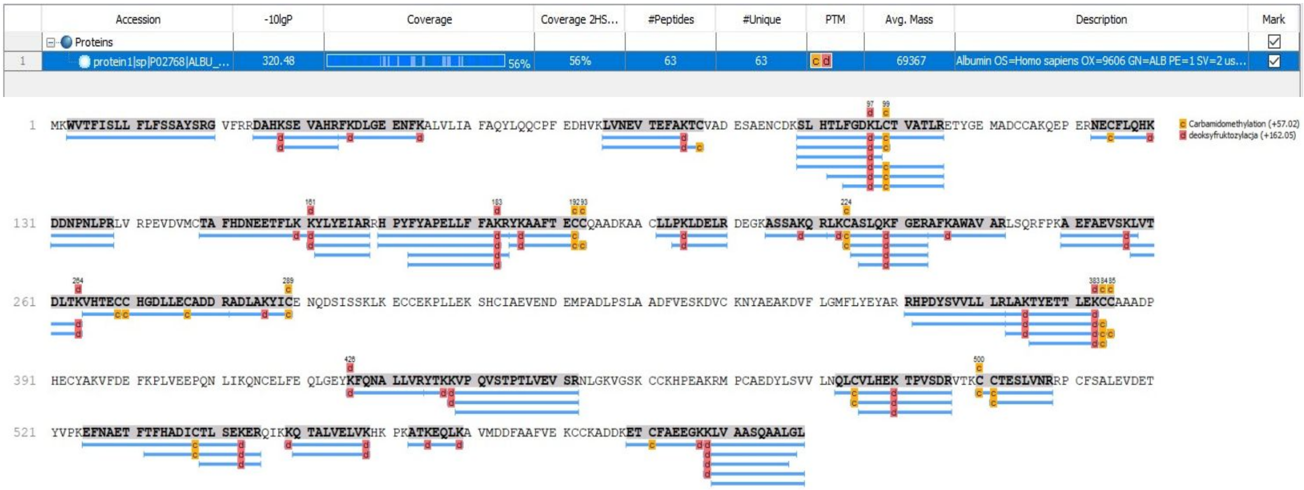

Figure S24 Sequence coverage of 56% based on LC-MS of glycated albumin hydrolysate after selective enrichment of sample by **TGR2** (only glycated peptides).

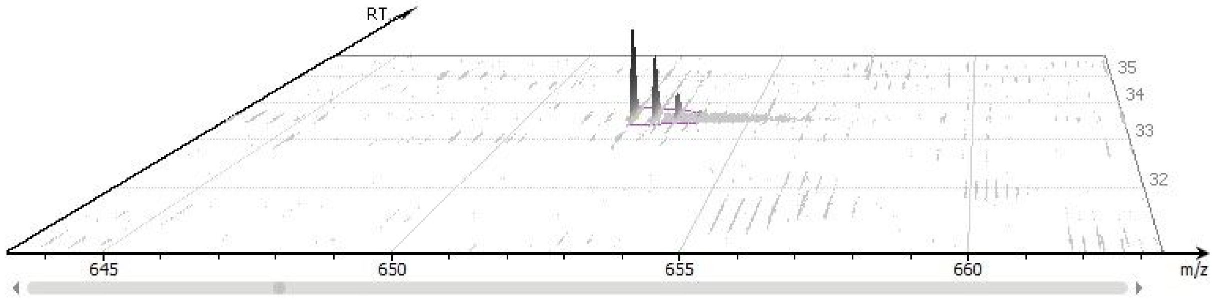

Figure S25 Graphical representation of correlation of intensity of the *m/z* signal to retention time of modified peptide (K*YLYEIAR) found in reacted fraction of glycated albumin hydrolysate after selective enrichment of sample by **TGR2**.

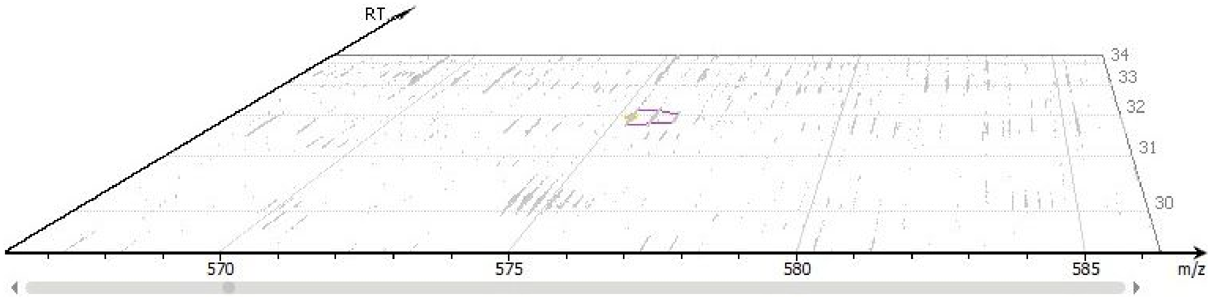

Figure S26 Graphical representation of correlation of intensity of the m/z signal to retention time of non-modified peptide (LVNEVTEFAK) found in reacted fraction of glycated albumin hydrolysate after selective enrichment of sample by **TGR2**.

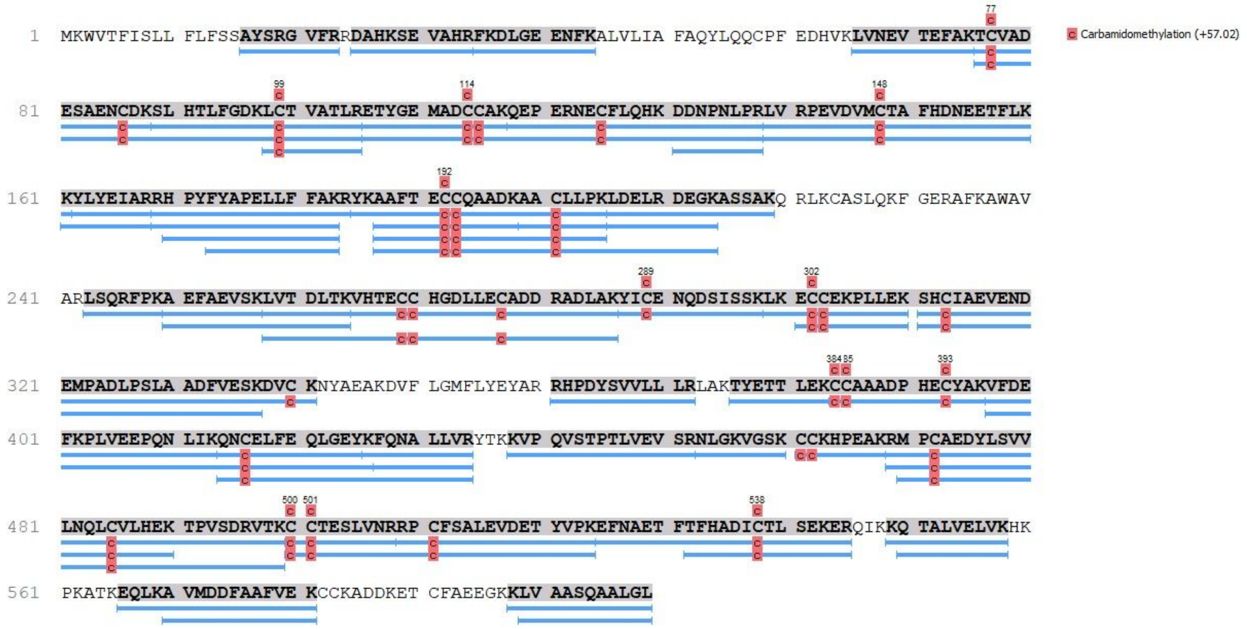

Figure S27 Sequence coverage of HSA based on LC-MS of patient's serum hydrolysate before selective enrichment of sample by **TGR2**.

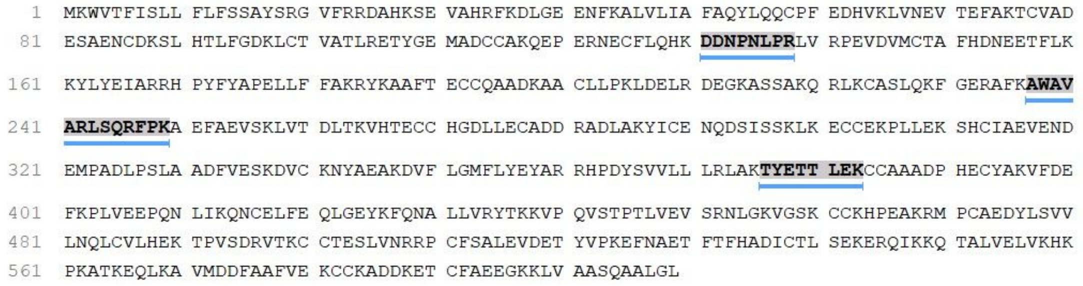

Figure S28 Sequence coverage of HSA based on LC-MS of patient's serum hydrolysate after selective enrichment of sample by **TGR2**.

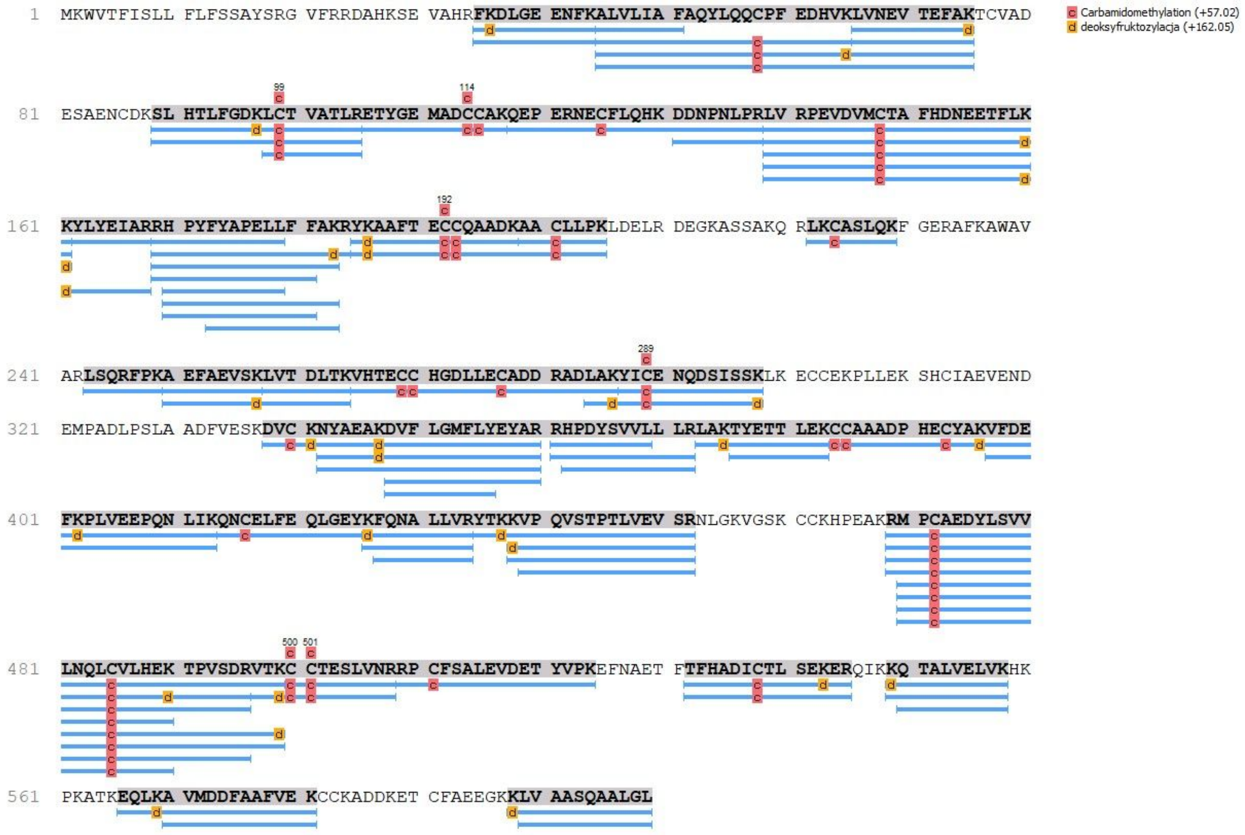

Figure S29 Sequence coverage of HSA based on LC-MS of glycated patient's serum hydrolysate before selective enrichment of sample by **TGR2**.

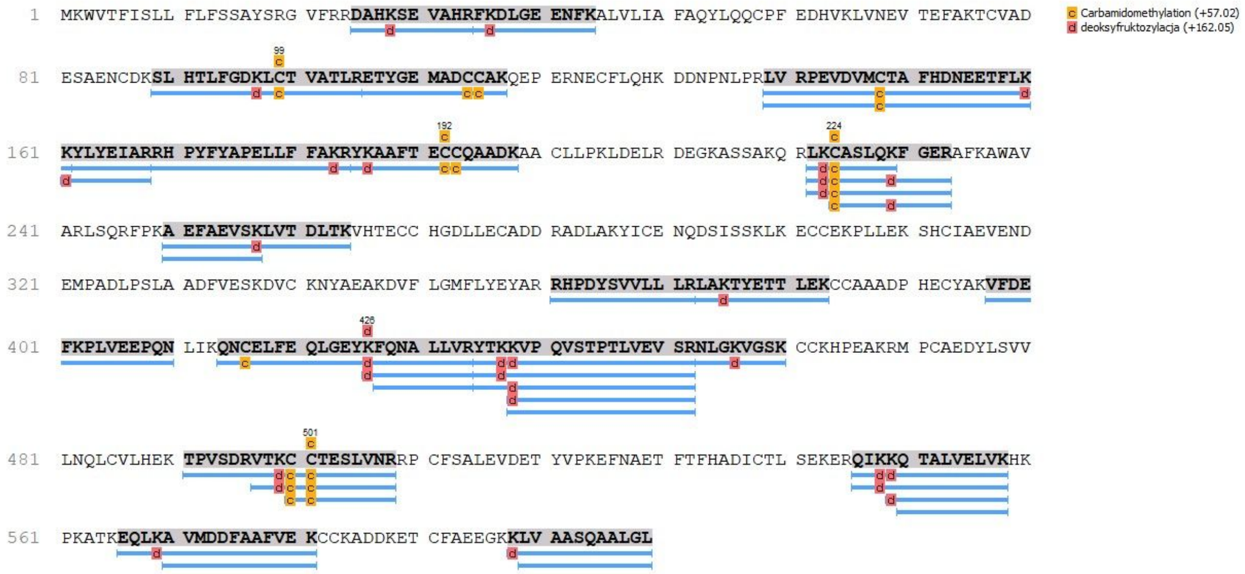

Figure S30 Sequence coverage of HSA based on LC-MS of glycated patient's serum hydrolysate after selective enrichment of sample by **TGR2**.

	Accession	-10lgP	Coverage	Coverage Cuk...	#Peptides	#Unique	PTM	Avg. Mass	Description	Mark
	Proteins									☑
1	P02768|ALBU_HUMAN	258.46	83%	83%	63	63	c	69367	Albumin OS=Homo sapiens OX=9606 GN=ALB PE=1 SV=2	☑
2	P00738|HPT_HUMAN	150.13	51%	51%	15	15	c	45205	Haptoglobin OS=Homo sapiens OX=9606 GN=HP PE=1 SV=1	☑
3	P01857|IGHG1_HUMAN	117.21	36%	36%	10	4	c	36106	Immunoglobulin heavy constant gamma 1 OS=Homo sapiens...	☑
4	P01009|A1AT_HUMAN	105.31	28%	28%	11	11		46737	Alpha-1-antitrypsin OS=Homo sapiens OX=9606 GN=SERPI...	☑
5	P01876|IGHA1_HUMAN	93.38	29%	29%	9	9	c	37655	Immunoglobulin heavy constant alpha 1 OS=Homo sapiens ...	☑
6	P02671|FIBA_HUMAN	90.45	13%	13%	10	10		94973	Fibrinogen alpha chain OS=Homo sapiens OX=9606 GN=FG...	☑
7	P02675|FIBB_HUMAN	90.01	33%	33%	10	10	c	55928	Fibrinogen beta chain OS=Homo sapiens OX=9606 GN=FGB ...	☑
8	P02679|FIBG_HUMAN	85.13	18%	18%	6	6	c	51512	Fibrinogen gamma chain OS=Homo sapiens OX=9606 GN=F...	☑
9	P02787|TRFE_HUMAN	83.40	20%	20%	9	9	c	77064	Serotransferrin OS=Homo sapiens OX=9606 GN=TF PE=1 S...	☑
10	P01834|IGKC_HUMAN	70.16	57%	57%	4	4	c	11765	Immunoglobulin kappa constant OS=Homo sapiens OX=9606...	☑
11	P01023|A2MG_HUMAN	60.11	4%	4%	5	5	c	163290	Alpha-2-macroglobulin OS=Homo sapiens OX=9606 GN=A2...	☑
12	P02647|APOA1_HUMAN	32.68	14%	14%	3	3		30778	Apolipoprotein A-I OS=Homo sapiens OX=9606 GN=APOA1 ...	☑
13	Q9UFH2|DYH17_HUMAN	32.49	0%	0%	3	3		509318	Dynein axonemal heavy chain 17 OS=Homo sapiens OX=96...	☑
14	O15018|PDZD2_HUMAN	28.76	1%	1%	3	3		301640	PDZ domain-containing protein 2 OS=Homo sapiens OX=960...	☑
15	Q6IQ32|ADNP2_HUMAN	20.40	1%	1%	2	2		122833	Activity-dependent neuroprotector homeobox protein 2 OS...	☑

Figure S31 Sequence coverage of human proteome based on LC-MS of patient's serum hydrolysate before selective enrichment of sample by **TGR2**.

	Accession	-10lgP	Coverage	Coverage Cuk...	#Peptides	#Unique	PTM	Avg. Mass	Description	Mark
	Proteins									☑
1	Q8NDH2|CC168_HUMAN	21.80	0%	0%	1	1		801931	Leucine-rich repeat transmembrane protein CCDC168 OS=H...	☑
2	Q6PIU2|NCEH1_HUMAN	21.46	2%	2%	1	1		45808	Neutral cholesterol ester hydrolase 1 OS=Homo sapiens OX...	☑
3	Q9NQW6|ANLN_HUMAN	20.58	1%	1%	1	1	c	124199	Anillin OS=Homo sapiens OX=9606 GN=ANLN PE=1 SV=2	☑

Figure S32 Sequence coverage of human proteome based on LC-MS of patient's serum hydrolysate after selective enrichment of sample by **TGR2**.

	Accession	-10lgP	Coverage	Coverage Cuk...	#Peptides	#Unique	PTM	Avg. Mass	Description	Mark
	Proteins									☑
1	P02768|ALBU_HUMAN	221.72	62%	62%	47	47	c d	69367	Albumin OS=Homo sapiens OX=9606 GN=ALB PE=1 SV=2	☑
2	P00738|HPT_HUMAN	111.19	20%	20%	9	9	c d	45205	Haptoglobin OS=Homo sapiens OX=9606 GN=HP PE=1 SV=1	☑
3	P02679|FIBG_HUMAN	75.84	11%	11%	4	4	c	51512	Fibrinogen gamma chain OS=Homo sapiens OX=9606 GN=F...	☑
4	P01876|IGHA1_HUMAN	64.60	21%	21%	5	5	c	37655	Immunoglobulin heavy constant alpha 1 OS=Homo sapiens ...	☑
5	P02675|FIBB_HUMAN	55.49	18%	18%	5	5	c	55928	Fibrinogen beta chain OS=Homo sapiens OX=9606 GN=FGB ...	☑
6	P01023|A2MG_HUMAN	51.47	3%	3%	4	4	c	163290	Alpha-2-macroglobulin OS=Homo sapiens OX=9606 GN=A2...	☑
7	P02671|FIBA_HUMAN	28.71	3%	3%	3	3		94973	Fibrinogen alpha chain OS=Homo sapiens OX=9606 GN=FG...	☑
8	Q9UFH2|DYH17_HUMAN	24.17	1%	1%	3	3		509318	Dynein axonemal heavy chain 17 OS=Homo sapiens OX=96...	☑
9	P01857|IGHG1_HUMAN	22.20	6%	6%	2	2		36106	Immunoglobulin heavy constant gamma 1 OS=Homo sapiens...	☑
10	P02787|TRFE_HUMAN	20.32	3%	3%	2	2	c	77064	Serotransferrin OS=Homo sapiens OX=9606 GN=TF PE=1 S...	☑

Figure S33 Sequence coverage of human proteome based on LC-MS of glycated patient's serum hydrolysate before selective enrichment of sample by **TGR2**.

	Accession	-10lgP	Coverage	Coverage Cuk...	#Peptides	#Unique	PTM	Avg. Mass	Description	Mark
	Proteins									☑
1	P02768|ALBU_HUMAN	151.44	25%	25%	16	16	c d	69367	Albumin OS=Homo sapiens OX=9606 GN=ALB PE=1 SV=2	☑
2	P02790|HEMO_HUMAN	28.37	3%	3%	1	1	d	51676	Hemopexin OS=Homo sapiens OX=9606 GN=HPX PE=1 SV=2	☑
3	P01023|A2MG_HUMAN	25.80	1%	1%	1	1	d	163290	Alpha-2-macroglobulin OS=Homo sapiens OX=9606 GN=A2...	☑
4	Q9BUF7|CRUM3_HUMAN	21.64	10%	10%	1	1		12854	Protein crumbs homolog 3 OS=Homo sapiens OX=9606 GN=...	☑

Figure S34 Sequence coverage of human proteome based on LC-MS of glycated patient's serum hydrolysate after selective enrichment of sample by **TGR2**.

Protein Group	Protein ID	Accession	-10lgP	Coverage (%)	Coverage (%) Protein+_GLC_H	Area Protein+_GLC_H	#Peptides	#Unique	#Spec Protein+_GLC_H	PTM	Avg. Mass	Description
1	4	P02662|CASA1_BOVIN	216.52	74	74	6.3356E6	45	30	1228	Y	24529	Alpha-S1-casein OS=Bos taurus OX=9913 GN=CSN1S1 PE=1 SV=2
3	1	P02754|LACB_BOVIN	205.00	88	88	3.396E6	31	8	1026	Y	19883	Beta-lactoglobulin OS=Bos taurus OX=9913 GN=LGB PE=1 SV=3
2	6	P02666|CASB_BOVIN	204.28	76	76	1.2777E7	41	41	1427	Y	25107	Beta-casein OS=Bos taurus OX=9913 GN=CSN2 PE=1 SV=2
6	5	P02663|CASA2_BOVIN	175.83	55	55	3.9663E6	27	27	526	Y	26019	Alpha-S2-casein OS=Bos taurus OX=9913 GN=CSN1S2 PE=1 SV=2
5	9	P02756|LACB_CAPHI	173.87	72	72	4.6414E4	24	1	632	Y	19976	Beta-lactoglobulin OS=Capra hircus OX=9925 GN=LGB PE=1 SV=2
7	14	P02668|CASK_BOVIN	158.25	54	54	1.7511E6	26	15	378	Y	21269	Kappa-casein OS=Bos taurus OX=9913 GN=CSN3 PE=1 SV=1
8	13	P00711|LALBA_BOVIN	157.66	73	73	1.2287E6	15	15	233	Y	16247	Alpha-lactalbumin OS=Bos taurus OX=9913 GN=LALBA PE=1 SV=2
9	3	P02769|ALBU_BOVIN	150.00	35	35	2.6337E5	20	20	134	Y	69294	Serum albumin OS=Bos taurus OX=9913 GN=ALB PE=1 SV=4
4	17	O62823|CASA1_BUBBU	128.83	39	39	0	17	2	603	Y	24327	Alpha-S1-casein OS=Bubalus bubalis OX=89462 GN=CSN1S1 PE=2 SV=2
11	11	P24627|TRFL_BOVIN	115.45	21	21	1.1815E5	14	12	84	Y	78056	Lactotransferrin OS=Bos taurus OX=9913 GN=LTF PE=1 SV=2
10	34	P50423|CASK_OREAM	106.99	27	27	2.4485E4	12	1	125	Y	21525	Kappa-casein OS=Oreamnos americanus OX=34873 GN=CSN3 PE=2 SV=1
12	33	P80195|GLCM1_BOVIN	78.89	54	54	3.7127E5	8	8	88	N	17152	Glycosylation-dependent cell adhesion molecule 1 OS=Bos taurus OX=9913 GN=GLYCAM1 PE=1 SV=2

Figure S35 Sequence coverage of identified milk protein of milk hydrolysate.

Protein Group	Protein ID	Accession	-10lgP	Coverage (%)	Coverage (%) Protein+_GLC_1_reacted	Area Protein+_GLC_1_reacted	#Peptides	#Unique	#Spec Protein+_GLC_1_reacted	PTM	Avg. Mass	Description
1	1	P02662|CASA1_BOVIN	143.86	38	38	1.0855E6	10	10	307	Y	24529	Alpha-S1-casein OS=Bos taurus OX=9913 GN=CSN1S1 PE=1 SV=2
2	6	P02666|CASB_BOVIN	138.98	27	27	5.2753E5	11	11	207	Y	25107	Beta-casein OS=Bos taurus OX=9913 GN=CSN2 PE=1 SV=2
4	5	P02754|LACB_BOVIN	110.93	39	39	3.5817E5	7	7	75	Y	19883	Beta-lactoglobulin OS=Bos taurus OX=9913 GN=LGB PE=1 SV=3
3	2	P02668|CASK_BOVIN	108.64	37	37	3.1808E5	9	9	152	Y	21269	Kappa-casein OS=Bos taurus OX=9913 GN=CSN3 PE=1 SV=1
5	11	P02663|CASA2_BOVIN	70.45	24	24	3.6217E5	5	5	34	Y	26019	Alpha-S2-casein OS=Bos taurus OX=9913 GN=CSN1S2 PE=1 SV=2

Figure S36 Sequence coverage of identified milk protein of reacted fraction after selective enrichment of sample by **TGR2**.

References

[1]Wołczański, G.; Płóciennik, H.; Lisowski, M.; Stefanowicz, P. A faster solid phase peptide synthesis method using ultrasonic agitation. *Tetrahedron Lett.* **2019**, *60*, 1814–1818, https://doi.org/10.1016/j.tetlet.2019.05.069

[2] Boratyński, J.; Roy, R.High temperature conjugation of proteins with carbohydrates. *Glycoconj. J.* **1998**, *15*, 131–138, https://doi.org/10.1023/A:1007067513242

[3] Kijewska, M.; Zawadzka, M.; Włodarczyk, K.; Stefanowicz, P. HPLC-free method of synthesis of isotopically labeled deoxyfructosylated peptides. *Anal. Bioanal. Chem.* **2022**, *414*, 3803-3811, https://doi.org/10.1007/s00216-022-04022-6

[4] Kijewska, M.; Zawadzka, M.; Stefanowicz, P. High-Temperature, Solid-Phase Reaction of α-Amino Groups in Peptides with Lactose and Glucose: An Alternative Mechanism Leading to an α-Ketoacyl Derivative. *J. Agric. Food Chem.* **2023**, *71*, 5796–5803, https://pubs.acs.org/doi/10.1021/acs.jafc.3c00821

[5] Waliczek, M.; Bąchor, R.; Kijewska, M.; Gąszczyk, D.; Panek-Laszczyńska, K.; Konieczny, A.; Dąbrowska, K.; Witkiewicz, W.; Marek-Bukowiec, K.; Tracz, J.; Łuczak, M;. Szewczuk, Z.; Stefanowicz, P. Isobaric duplex based on a combination of $^{16}O/^{18}O$ enzymatic exchange and labeling with pyrylium salts. *Anal. Chim. Acta*. **2019**, *1048*, 96-104, https://doi.org/10.1016/j.aca.2018.10.012

[6] Stefanowicz, P.; Kijewska, M.; Kluczyk, A.; Szewczuk, Z. Detection of glycation sites in proteins by high-resolution mass spectrometry combined with isotopic labeling. *Anal. Biochem.* **2010**, *400*, 237-43, https://doi.org/10.1016/j.ab.2010.02.011

[7] Soboleva, A.; Modzel, M.; Didio, A.; Płóciennik, H.; Kijewska, M.; Grischina, T.; Karonova, T.; Bilova, T.; Stefanov, V.; Stefanowicz, P.; Frolov, A. Quantification of prospective type 2 diabetes mellitus biomarkers by stable isotope dilution with bi-labeled standard glycated peptides. *Anal. Methods.* **2017**, *9*, 409–418, https://doi.org/10.1039/C6AY02483A

[8] Greifenhagen, U.; Nguyen, V.D.; Moschner, J.; Giannis, A.; Frolov, A.; Hoffmann, R. Sensitive and site-specific identification of carboxymethylated and carboxyethylated peptides in tryptic digests of proteins and human plasma. *J. Proteome Res.* **2015**, *14*, 768-777, https://doi.org/10.1021/pr500799m
